# Associations of insulin resistance surrogates with cardiovascular disease in early stage cardiovascular–kidney–metabolic syndrome: a prospective cohort study

**DOI:** 10.3389/fcvm.2026.1876121

**Published:** 2026-10-20

**Authors:** Ao Li, Shiyu Tian, Jianjun Zhang, Dong Zhang, Jie Xu, Xianyun Wang, Hongxuan Yan, Xiaohui Sun, Feng Ning, Lei Zhang

**Affiliations:** 1 Department of Epidemiology and Health Statistics, Qingdao Medical College, Qingdao University, Qingdao, Shandong, China; 2 Qingdao Centers for Disease Control and Prevention, Qingdao, Shandong, China; 3 Qingdao Municipal Health Big Data Center, Qingdao, Shandong, China; 4 Huangdao Centers for Disease Control and Prevention, Qingdao, Shandong, China; 5 Department of Rehabilitation Medicine, Qingdao Municipal Hospital, Qingdao, Shandong, China; 6 Qingdao Binhai Medical College, Qingdao, Shandong, China; 7 Qingdao Prevention Medicine Institute, Qingdao, Shandong, China; 8 Qingdao Endocrine and Diabetes Hospital, Qingdao, Shandong, China

**Keywords:** cardiovascular diseases, cardiovascular-kidney-metabolic syndrome, insulin resistance surrogates, prospective cohort studies, risk assessment

## Abstract

**Background:**

The early stages 0–3 of cardiovascular–kidney–metabolic (CKM) syndrome represent a critical window for cardiovascular disease (CVD) prevention. Although insulin resistance (IR) is a key driver of CVD risk, systematic comparisons of IR surrogates in CVD remain limited. This study examined the associations of 12 IR surrogates with CVD in participants with stages 0–3 CKM syndrome.

**Methods:**

This prospective study included 5,625 participants with stages 0–3 CKM syndrome from the Qingdao Diabetes Program. Twelve IR surrogates related to CVD were evaluated using multivariable-adjusted Cox models, restricted cubic splines, sensitivity analyses, and predictive performance analyses, such as triglyceride–glucose index, atherogenic index of plasma, cardiometabolic index, metabolic score for IR (METS-IR), cholesterol-high-density lipoprotein-glucose (CHG) index, CHG plus body mass index, CHG plus waist circumference, CHG plus waist-to-height ratio, CHG plus body roundness index, CHG plus weight-adjusted waist index, CHG plus A body shape index, and CHG plus Chinese visceral adiposity index (CVAI).

**Results:**

Over a median follow-up of 15.2 years, 1,407 incident CVD events occurred. In multivariable-adjusted analyses, all 12 IR surrogates were significantly associated with CVD risk (all *P* < 0.05), of CHG-CVAI showing the best performance (Q4 vs. Q1: HR = 2.10, 95% CI = 1.74–2.52). Most indices showed nonlinear dose–response relationships. The associations were higher risk for adults aged <60 years and urban residents as compared with counterparts, and sensitivity analyses confirmed the robustness of the primary findings. Furthermore, addition of each IR surrogate improved CVD risk prediction: C-index ranging from 0.669 to 0.674, NRI ranging from 0.089 to 0.205 and IDI ranging from 0.0004 to 0.0032 (all *P* < 0.001).

**Conclusion:**

All IR surrogates, particularly CHG-CVAI were independently associated with incident CVD in early-stages CKM syndrome. These simple, low-cost indices improved risk stratification and moderately identify CVD individuals in high risk population.

## Introduction

Cardiovascular–kidney–metabolic (CKM) syndrome, a construct proposed by the American Heart Association in 2023 (1, 2), represents a complex interplay between metabolic disturbances, chronic kidney disease (CKD), and cardiovascular disease (CVD). Because of its high prevalence, CKM syndrome imposes a substantial global public health burden (3). According to the AHA, the early, predominantly asymptomatic stages (0–3) of CKM syndrome represent a critical window for CVD prevention, particularly because timely interventions during this phase may halt progression toward overt clinical outcomes (2).

Insulin resistance (IR) is a core pathological mechanism that links metabolic dysfunction to atherosclerotic CVD (4). Because the gold-standard technique of hyperinsulinemic-euglycemic clamping is invasive and unsuitable for routine use, simple IR surrogates are widely used in clinical practice (5). The triglyceride-glucose (TyG) index is a well-validated marker of IR and has been consistently associated with elevated CVD risk (6). Similarly, the atherogenic index of plasma (AIP), cardiometabolic index (CMI), and metabolic score for IR (METS-IR) have emerged as promising surrogates reflecting dyslipidemia, visceral adiposity, and systemic metabolic dysregulation (7–9). The cholesterol-high-density lipoprotein-glucose (CHG) index combines total cholesterol (TC), high-density lipoprotein cholesterol (HDL-C), and fasting blood glucose (FBG) to reflect both lipid and glucose metabolism (10). Because excess adiposity is closely associated with metabolic disorders and CVD, the CHG index has been further combined with various obesity indicators to derive a set of novel composite indices (11).

Although studies have reported associations between IR surrogates and CVD in general or specific populations (12, 13), relevant evidence pertaining to Chinese adults in early CKM syndrome stages 0–3 remains limited. Moreover, few studies have comprehensively evaluated the predictive performance of IR surrogates. In the present prospective cohort study based on the Qingdao Diabetes Program with a median follow-up of 15.2 years, we systematically investigated the associations of 12 IR surrogates with incident CVD in participants with stage 0–3 CKM syndrome and their potential value for refining CVD risk stratification.

## Methods

### Study cohort

This study was based on the Qingdao Diabetes Program (14, 15). Two independent population-based cross-sectional diabetes surveys were conducted in 2006 and 2009. In both surveys, a unified standardized protocol was adopted, and stratified random cluster sampling was applied to recruit representative residents aged 35–74 years with at least 5 years of residency in Qingdao. The 2006 and 2009 cohorts comprised 5,355 and 5,165 non-overlapping individuals. As shown in Figure 1, a pool analysis in current study including 1,294 individuals from the 2006 survey followed up in 2009 and 5,165 individuals in 2009 survey. After excluding 39 participants without ID, 518 participants with prevalent CVD, 277 participants because of incomplete data on covariables required, the present study comprises 2,192 men and 3,433 women.

**Figure 1:**
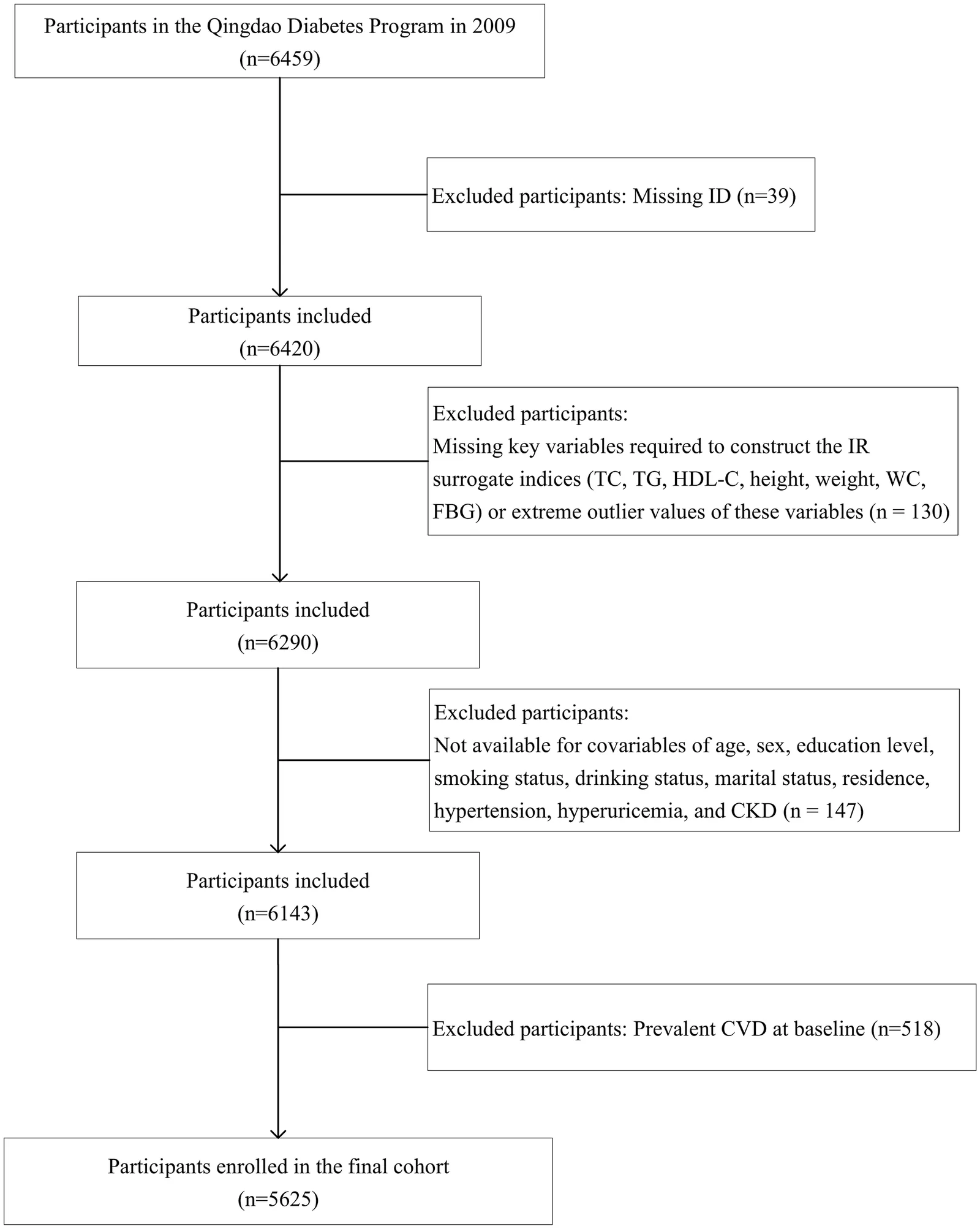
Flow chart of participant selection.

### Definitions of CKM syndrome stages 0–3

CKM syndrome stages were defined on the basis of the 2023 American Heart Association Presidential Advisory (2). Stage 0 was defined as the absence of risk factors for CKM syndrome. Stage 1 was defined as the presence of excess or dysfunctional adiposity (e.g., overweight or obesity, abdominal obesity, or prediabetes). Stage 2 was defined as the presence of metabolic risk factors (e.g., type 2 diabetes, hypertension, or hypertriglyceridemia) or CKD. Stage 3 was defined as subclinical cardiovascular disease in individuals with excess or dysfunctional adiposity, metabolic risk factors, or CKD (e.g., an elevated 10-year CVD risk indicated by the Framingham risk score [≥21.5 for women and ≥21.6 for men]) (16), or very-high-risk CKD indicated by an eGFR of <30 mL/min/1.73 m^2^ (17). eGFR was calculated using the 2021 CKD-EPI creatinine equation without a race coefficient (18): eGFR = 142 × min(SCr/*κ*, 1)^*α* × max(SCr/*κ*, 1)^(−1.200) × 0.9938^Age×1.012 [if female], where SCr is serum creatinine (mg/dL), *κ* is 0.7 (women) or 0.9 (men), and *α* is −0.241 (women) or −0.302 (men). Renal evaluation and CKD staging strictly followed the Kidney Disease: Improving Global Outcomes guidelines (19). The detailed diagnostic criteria for stage 0–3 CKM syndrome are provided in Supplementary Table S1.

### Exposure and outcome variables

Fasting venous blood samples were collected for biochemical testing. All biomarkers were measured using enzymatic methods. Standardized anthropometric measurements were conducted by uniformly trained and certified health technicians under strict quality control conditions. On the basis of baseline data, 12 IR surrogates were calculated, with their formulas detailed in Supplementary Table S2: CHG index, CHG plus body mass index (BMI), CHG plus WC, CHG plus waist-to-height ratio (WHtR), CHG plus body roundness index (BRI), CHG plus A body shape index (ABSI), CHG plus weight-adjusted waist index (WWI), CHG plus Chinese visceral adiposity index (CVAI), TyG index, METS-IR, CMI, and AIP.

The primary outcome was incident CVD during follow up in participants with stages 0–3 CKM syndrome, defined as the first occurrence of a major cardiac event or stroke. CVD outcomes were ascertained using data from the Qingdao Cardiovascular Disease Surveillance System. Follow-up started from the 2009 examination and extended until the first CVD event, death, loss to follow-up, or January 31, 2026, whichever occurred first; participants without an event were censored at the earliest of these dates. In accordance with the *International Classification of Diseases*, *Tenth Revision* guidelines, CVD outcomes comprised coronary artery disease (codes I20–I25.9), hemorrhagic stroke (I60-I61), ischemic stroke (codes I63–I64) and other types of stroke (I62).

### Covariates

On the basis of clinical experience and the literature, we selected the following potential confounders: (1) demographic characteristics—age, sex, education level (no formal education, primary school, middle school, or high school and above), marital status (married or others), and residence (urban or rural); (2) lifestyle factors—smoking status (current, former, or never) and drinking status (current, former, or never); and (3) clinical characteristics—hypertension, hyperuricemia and CKD. CKD was defined as a prior clinical diagnosis of CKD or an eGFR of <60 mL/min/1.73 m^2^ (19). Hypertension was defined as a physician-diagnosed history, systolic blood pressure ≥140 mmHg, or diastolic blood pressure ≥90 mmHg (20). Hyperuricemia was defined as serum uric acid level ≥ 420 μmol/L for men and ≥ 360 μmol/L for women (21).

### Statistical analysis

Baseline characteristics were summarized by outcome occurrence and CKM syndrome stage. Normally distributed continuous variables are presented as mean ± standard deviation values, and skewed variables are presented as median (interquartile range) values. Categorical variables are presented as frequencies (percentages). Intergroup comparisons were conducted using *t*, Mann–Whitney *U*, Kruskal–Wallis, or chi-square tests, as appropriate. Spearman's rank correlation analysis was performed to identify the associations between IR surrogates.

Each index was analyzed both as a continuous variable and by quartiles with Cox proportional-hazards models to identify its association with incident CVD in participants with stages 0–3 CKM syndrome, with hazard risk and 95% confidence interval. Model 1 was not adjustment. Model 2 was adjusted for age, sex, residence, smoking status, drinking status, marital status, and education level. Model 3 was additionally adjusted for hypertension, hyperuricemia, and CKD. We computed generalized variance inflation factors for each single-index Model 3 to assess multicollinearity. The Benjamini–Hochberg false discovery rate (FDR) correction was applied to the *P* for trend values to evaluate multiple testing across the 12 indices. The proportional-hazards assumption was tested using Schoenfeld residuals; for indices with a significant time-interaction term, additional sensitivity analyses stratified by follow-up duration (≤8 vs. > 8 years). The further data analysis was attached to the Supplementary Table S3-S5.

Restricted cubic spline (RCS) regression analysis was performed to explore the dose–response relationships of individual IR surrogates with CVD risk, with four knots at the 5th, 35th, 65th, and 95th percentiles and the median as the reference. If nonlinearity was detected (*P* for nonlinearity < 0.05), two-piecewise Cox models were fitted to identify threshold effects, with inflection points identified by a recursive algorithm and compared against a linear model using the likelihood ratio test. The robustness was assessed by 1,000-replicate bootstrap resampling.

Subgroup analyses by age (<60 vs. ≥ 60 years), sex, residence, CKM syndrome stage (0 or 1 vs. 2 or 3), hypertension and hyperuricemia were performed. Interaction effects were tested using the likelihood ratio test. Sensitivity analyses included restricting the sample to participants with a follow-up duration of ≥5 years, excluding those on lipid-lowering or hypoglycemic medications, and analyzing indices by tertiles. We also performed Fine-Gray subdistribution hazard regression to assess potential competing risk, with non-cardiovascular death as the competing event.

The predictive performances of the IR surrogates were evaluated using the Harrell's C-index and time-dependent area under curve (AUC) at 5, 10, and 15 years, together with NRI and IDI analyses at 10 years (22). The 95% CIs and *P*-values for NRI and IDI were derived from 500 bootstrap resamples, in which the predicted 10-year risks from the two Cox models were held fixed and the NRI/IDI statistics were recomputed for each resample, and *P*-values were calculated as twice the smaller one-sided tail probability. Calibration of the basic model was assessed using a calibration plot comparing predicted and observed 10-year CVD-free survival based on 100 bootstrap resamples. All statistical analyses were performed using R software (version 4.2.2). All tests were two-sided, and *P* < 0.05 was considered statistically significant.

## Results

### Baseline characteristics

This study included 5,625 participants with stage 0–3 CKM syndrome (mean age: 51.98 ± 10.40 years; women, 61.0%). Because of the small number of participants in stage 3 (*n* = 18), stage 3 was combined with stage 2 for the present analysis. With increasing CKM stage (stage 0, stage 1, and stage 2–3), age and continuous anthropometric and metabolic parameters—including BMI, WC, WHtR, BRI, ABSI, CVAI, FBG, TC, TG, UA, SBP, DBP, and the Framingham risk score—as well as all 12 IR surrogates increased progressively, whereas HDL-C and eGFR decreased progressively (all *P* < 0.01). The distributions of the categorical factors (sex, marital status, education level, residence, smoking status, and drinking status) also differed significantly across CKM stages (all *P* < 0.05). Moreover, the overall incidence of CVD gradually increased with increasing disease severity (*P* < 0.001).

Over a median follow-up of 15.2 years, 1,407 participants (25.0%) developed incident CVD. Participants with incident CVD were older than those without it. The CVD group had a lower level of education and a higher prevalence of hypertension, dyslipidemia, hyperuricemia, and diabetes than did the no-CVD group (all *P* < 0.001). Regarding anthropometric and laboratory parameters, weight, BMI, BRI, WC, WHtR, CVAI, SBP, DBP, FBG, TC, and TG were higher and HDL-C was lower in the CVD group than in the no-CVD group (all *P* < 0.001). Notably, all 12 IR surrogates were significantly higher in the CVD group than in the no-CVD group (all *P* < 0.001). Tables 1, 2 present baseline characteristics summarized by CKM syndrome stage and incident CVD status, respectively.

**Table 1:** Baseline characteristics by stage of CKM stage 0–3.

Characteristic	Total (*n* = 5,625)	Stage 0 (*n* = 359)	Stage 1 (*n* = 640)	Stage 2–3 (*n* = 4,626)	*P*
Sex					<0.001
Men	2,192 (39.0)	137 (38.2)	191 (29.8)	1,864 (40.3)	
Women	3,433 (61.0)	222 (61.8)	449 (70.2)	2,762 (59.7)	
Age (years)	51.98 (10.40)	46.74 (9.19)	47.42 (8.95)	53.02 (10.39)	<0.001
Marital status					0.005
Married	5,309 (94.4)	350 (97.5)	613 (95.8)	4,346 (93.9)	
vOther	316 (5.6)	9 (2.5)	27 (4.2)	280 (6.1)	
Education level					<0.001
No formal education	1,424 (25.3)	53 (14.8)	154 (24.1)	1,217 (26.3)	
Primary school	1,469 (26.1)	81 (22.6)	162 (25.3)	1,226 (26.5)	
Middle school	1,834 (32.6)	148 (41.2)	230 (35.9)	1,456 (31.5)	
High school or above	898 (16.0)	77 (21.4)	94 (14.7)	727 (15.7)	
Smoking status					0.007
Current	1,395 (24.8)	109 (30.4)	153 (23.9)	1,133 (24.5)	
Never	3,979 (70.7)	239 (66.6)	470 (73.4)	3,270 (70.7)	
Former	251 (4.5)	11 (3.1)	17 (2.7)	223 (4.8)	
Drinking status					0.002
Current	1,806 (32.1)	128 (35.7)	166 (25.9)	1,512 (32.7)	
Never	3,734 (66.4)	226 (63.0)	468 (73.1)	3,040 (65.7)	
Former	85 (1.5)	5 (1.4)	6 (0.9)	74 (1.6)	
Residence					<0.001
Urban	1,473 (26.2)	100 (27.9)	127 (19.8)	1,246 (26.9)	
Rural	4,152 (73.8)	259 (72.1)	513 (80.2)	3,380 (73.1)	
Height (cm)	162.06 (8.02)	162.38 (7.63)	161.77 (7.47)	162.08 (8.12)	0.427
Weight (kg)	66.29 (11.28)	56.01 (6.60)	64.05 (9.05)	67.40 (11.39)	<0.001
BMI (kg/m^2^)	25.20 (3.62)	21.21 (1.68)	24.48 (3.06)	25.61 (3.60)	<0.001
BRI	3.82 (1.29)	2.50 (0.62)	3.48 (1.06)	3.97 (1.29)	<0.001
WC (cm)	84.15 (10.31)	73.02 (6.23)	81.30 (8.42)	85.40 (10.21)	<0.001
WWI	10.37 (0.94)	9.78 (0.72)	10.19 (0.92)	10.44 (0.93)	<0.001
WHtR	0.52 (0.06)	0.45 (0.04)	0.50 (0.05)	0.53 (0.06)	<0.001
ABSI	0.08 (0.01)	0.07 (0.01)	0.08 (0.01)	0.08 (0.01)	<0.001
CVAI	86.35 (42.06)	37.12 (23.55)	65.79 (30.02)	93.01 (41.14)	<0.001
Hypertension					<0.001
No	2,952 (52.5)	359 (100.0)	640 (100.0)	1,953 (42.2)	
Yes	2,673 (47.5)	0 (0.0)	0 (0.0)	2,673 (57.8)	
Dyslipidemia					<0.001
No	3,876 (68.9)	322 (89.7)	545 (85.2)	3,009 (65.0)	
Yes	1,749 (31.1)	37 (10.3)	95 (14.8)	1,617 (35.0)	
Hyperuricemia					<0.001
No	4,892 (87.0)	338 (94.2)	597 (93.3)	3,957 (85.5)	
Yes	733 (13.0)	21 (5.8)	43 (6.7)	669 (14.5)	
SBP (mmHg)	133.83 (21.64)	110.41 (9.81)	113.87 (9.23)	138.41 (20.77)	<0.001
DBP (mmHg)	82.40 (11.86)	68.96 (6.03)	70.19 (5.41)	85.14 (11.04)	<0.001
FBG (mmol/L)	5.91 (1.49)	5.03 (0.39)	5.57 (0.51)	6.02 (1.60)	<0.001
TC (mmol/L)	5.29 (1.03)	4.95 (0.96)	5.05 (0.89)	5.35 (1.05)	<0.001
TG (mmol/L)	1.48 (1.19)	0.86 (0.29)	0.93 (0.32)	1.60 (1.27)	<0.001
HDL-C (mmol/L)	1.63 (0.38)	1.79 (0.34)	1.72 (0.33)	1.60 (0.38)	<0.001
UA (μmol/L)	304.91 (80.81)	274.84 (71.24)	282.88 (66.64)	310.29 (82.26)	<0.001
Diabetes					<0.001
No	4,777 (84.9)	359 (100.0)	640 (100.0)	3,778 (81.7)	
Yes	848 (15.1)	0 (0.0)	0 (0.0)	848 (18.3)	
Chronic kidney disease					0.004
No	5,574 (99.1)	359 (100.0)	640 (100.0)	4,575 (98.9)	
Yes	51 (0.9)	0 (0.0)	0 (0.0)	51 (1.1)	
eGFR (mL/min/1.73 m^2^)	108.84 (10.38)	112.48 (8.98)	112.37 (8.27)	108.07 (10.58)	<0.001
Framingham risk score	10.30 (5.01)	6.44 (4.67)	6.94 (4.57)	11.07 (4.75)	<0.001
CHG	5.14 (0.34)	4.83 (0.23)	4.99 (0.23)	5.19 (0.35)	<0.001
CHG-BMI	129.98 (23.11)	102.44 (9.99)	122.19 (16.52)	133.20 (22.94)	<0.001
CHG-BRI	19.80 (7.26)	12.07 (3.12)	17.38 (5.42)	20.73 (7.27)	<0.001
CHG-WC	434.01 (69.42)	352.77 (36.47)	405.99 (48.14)	444.19 (68.64)	<0.001
CHG-WWI	53.41 (6.61)	47.24 (4.25)	50.88 (5.28)	54.24 (6.59)	<0.001
CHG-WHtR	2.68 (0.43)	2.17 (0.22)	2.51 (0.31)	2.74 (0.43)	<0.001
CHG-ABSI	0.40 (0.05)	0.36 (0.03)	0.38 (0.04)	0.40 (0.05)	<0.001
CHG-CVAI	451.69 (234.93)	180.76 (116.30)	330.49 (155.89)	489.49 (231.41)	<0.001
TyG	8.65 (0.63)	8.09 (0.35)	8.27 (0.36)	8.74 (0.63)	<0.001
METS-IR	7.60 (0.37)	7.16 (0.15)	7.40 (0.18)	7.66 (0.37)	<0.001
AIP	−0.11 (0.30)	−0.33 (0.18)	−0.28 (0.19)	−0.07 (0.30)	<0.001
CMI	0.55 (0.66)	0.23 (0.10)	0.29 (0.13)	0.61 (0.71)	<0.001
CVD					<0.001
No	4,218 (75.0)	301 (83.8)	527 (82.3)	3,390 (73.3)	
Yes	1,407 (25.0)	58 (16.2)	113 (17.7)	1,236 (26.7)	

Data are presented as mean (SD) or *n* (percentages).

*P*-values were derived from the Kruskal–Wallis test for continuous variables and the chi-square test for categorical variables.

**Table 2:** Baseline characteristics by incident CVD during follow-up.

Characteristic	Total (*n* = 5,625)	Non-CVD (*n* = 4,218)	CVD (*n* = 1,407)	*P*
Age (years)	51.98 (10.40)	50.98 (10.45)	54.96 (9.68)	<0.001
Height (cm)	162.06 (8.02)	162.10 (8.01)	161.95 (8.06)	0.525
Weight (kg)	66.29 (11.28)	65.74 (11.22)	67.92 (11.30)	<0.001
BMI (kg/m^2^)	25.20 (3.62)	24.98 (3.59)	25.86 (3.64)	<0.001
BRI	3.82 (1.29)	3.73 (1.27)	4.10 (1.31)	<0.001
WC (cm)	84.15 (10.31)	83.40 (10.22)	86.39 (10.25)	<0.001
WWI	10.37 (0.94)	10.32 (0.94)	10.52 (0.90)	<0.001
WHtR	0.52 (0.06)	0.52 (0.06)	0.53 (0.06)	<0.001
ABSI	0.08 (0.01)	0.08 (0.01)	0.08 (0.01)	<0.001
CVAI	86.35 (42.06)	81.86 (41.63)	99.81 (40.45)	<0.001
SBP (mmHg)	133.83 (21.64)	132.34 (21.14)	138.29 (22.50)	<0.001
DBP (mmHg)	82.40 (11.86)	81.63 (11.56)	84.71 (12.42)	<0.001
FBG (mmol/L)	5.91 (1.49)	5.86 (1.45)	6.06 (1.59)	<0.001
TC (mmol/L)	5.29 (1.03)	5.23 (1.01)	5.47 (1.06)	<0.001
TG (mmol/L)	1.48 (1.19)	1.43 (1.18)	1.62 (1.19)	<0.001
HDL-C (mmol/L)	1.63 (0.38)	1.64 (0.38)	1.58 (0.37)	<0.001
UA (μmol/L)	304.91 (80.81)	302.18 (80.64)	313.08 (80.78)	<0.001
eGFR (mL/min/1.73 m^2^)	108.84 (10.38)	109.62 (10.17)	106.48 (10.64)	<0.001
CHG	5.14 (0.34)	5.12 (0.34)	5.22 (0.34)	<0.001
CHG-BMI	129.98 (23.11)	128.16 (22.81)	135.43 (23.14)	<0.001
CHG-WC	434.01 (69.42)	427.89 (68.43)	452.37 (69.14)	<0.001
CHG-CVAI	451.69 (234.93)	426.27 (231.35)	527.89 (229.11)	<0.001
CHG-BRI	19.80 (7.26)	19.22 (7.11)	21.54 (7.40)	<0.001
CHG-WHtR	2.68 (0.43)	2.64 (0.42)	2.80 (0.43)	<0.001
CHG-WWI	53.41 (6.61)	52.88 (6.58)	54.99 (6.47)	<0.001
CHG-ABSI	0.40 (0.05)	0.39 (0.05)	0.41 (0.05)	<0.001
TyG	8.65 (0.63)	8.60 (0.63)	8.78 (0.62)	<0.001
METS-IR	7.60 (0.37)	7.57 (0.37)	7.68 (0.36)	<0.001
AIP	−0.11 (0.30)	−0.13 (0.30)	−0.05 (0.29)	<0.001
CMI	0.55 (0.66)	0.52 (0.68)	0.62 (0.58)	<0.001
Sex				0.064
Men	2,192 (39.0)	1,673 (39.7)	519 (36.9)	
Women	3,433 (61.0)	2,545 (60.3)	888 (63.1)	
Marital status				0.692
Married	5,309 (94.4)	3,984 (94.5)	1,325 (94.2)	
Other	316 (5.6)	234 (5.5)	82 (5.8)	
Education level				<0.001
No formal education	1,424 (25.3)	983 (23.3)	441 (31.3)	
Primary school	1,469 (26.1)	1,150 (27.3)	319 (22.7)	
Middle school	1,834 (32.6)	1,428 (33.9)	406 (28.9)	
High school or above	898 (16.0)	657 (15.6)	241 (17.1)	
Smoking status				0.126
Current	1,395 (24.8)	1,072 (25.4)	323 (23.0)	
Never	3,979 (70.7)	2,965 (70.3)	1,014 (72.1)	
Former	251 (4.5)	181 (4.3)	70 (5.0)	
Drinking status				<0.001
Current	1,806 (32.1)	1,401 (33.2)	405 (28.8)	
Never	3,734 (66.4)	2,762 (65.5)	972 (69.1)	
Former	85 (1.5)	55 (1.3)	30 (2.1)	
Residence				<0.001
Urban	1,473 (26.2)	987 (23.4)	486 (34.5)	
Rural	4,152 (73.8)	3,231 (76.6)	921 (65.5)	
Hypertension				<0.001
No	2,952 (52.5)	2,364 (56.0)	588 (41.8)	
Yes	2,673 (47.5)	1,854 (44.0)	819 (58.2)	
Dyslipidemia				<0.001
No	3,876 (68.9)	3,044 (72.2)	832 (59.1)	
Yes	1,749 (31.1)	1,174 (27.8)	575 (40.9)	
Hyperuricemia				<0.001
No	4,892 (87.0)	3,701 (87.7)	1,191 (84.6)	
Yes	733 (13.0)	517 (12.3)	216 (15.4)	
Diabetes				<0.001
No	4,777 (84.9)	3,642 (86.3)	1,135 (80.7)	
Yes	848 (15.1)	576 (13.7)	272 (19.3)	
Chronic kidney disease				0.168
No	5,574 (99.1)	4,184 (99.2)	1,390 (98.8)	
Yes	51 (0.9)	34 (0.8)	17 (1.2)	

Data are presented as mean (SD) or *n* (percentages).

*P*-values were derived from the Kruskal–Wallis test for continuous variables and the chi-square test for categorical variables.

### Associations of IR surrogates With CVD risk in stages 0–3 CKM syndrome

The associations of the 12 IR surrogates with incident CVD are shown in Table 3. In Model 3, all 12 IR surrogates were significantly associated with CVD risk across the quartiles (all *P* for trend < 0.001). CHG-CVAI had the highest Q4 HR (HR = 2.10, 95% CI = 1.74–2.52), followed by METS-IR (HR = 1.87, 95% CI = 1.58–2.21) and CHG-BMI (HR = 1.81, 95% CI = 1.54–2.14). When analyzed as continuous variables, all indices showed significant positive associations with CVD risk; the per-1-SD HR was highest for CHG-CVAI (HR = 1.24, 95% CI = 1.17–1.31) and lowest for CMI (HR = 1.07, 95% CI = 1.03–1.11).

**Table 3:** Association between IR surrogates and CVD incidence in individuals with CKM syndrome stages 0–3.

	CVD Events,n	Model 1		Model 2		Model 3	
		HR (95%CI)	*P*	HR (95%CI)	*P*	HR (95%CI)	*P*
CHG							
per 1 SD	1,407	1.32 (1.26,1.38)	<0.001	1.23 (1.17, 1.29)	<0.001	1.20 (1.14, 1.26)	<0.001
Q1	228	Ref		Ref		Ref	
Q2	301	1.38 (1.16,1.64)	<0.001	1.25 (1.06, 1.49)	0.010	1.22 (1.03, 1.46)	0.022
Q3	414	2.03 (1.72,2.38)	<0.001	1.74 (1.48, 2.05)	<0.001	1.66 (1.41, 1.96)	<0.001
Q4	464	2.39 (2.04,2.80)	<0.001	1.90 (1.61, 2.23)	<0.001	1.76 (1.49, 2.08)	<0.001
*P*-trend		<0.001		<0.001		<0.001	
CHG-BMI							
per 1 SD	1,407	1.30 (1.24,1.37)	<0.001	1.25 (1.19, 1.32)	<0.001	1.21 (1.15, 1.28)	<0.001
Q1	235	Ref		Ref		Ref	
Q2	336	1.46 (1.23,1.72)	<0.001	1.38 (1.16, 1.63)	<0.001	1.32 (1.12, 1.57)	0.001
Q3	364	1.66 (1.41,1.95)	<0.001	1.52 (1.29, 1.79)	<0.001	1.43 (1.21, 1.69)	<0.001
Q4	472	2.26 (1.93,2.64)	<0.001	2.00 (1.70, 2.34)	<0.001	1.81 (1.54, 2.14)	<0.001
*P*-trend		<0.001		<0.001		<0.001	
CHG-WC							
per 1 SD	1,407	1.37 (1.30,1.44)	<0.001	1.25 (1.19, 1.32)	<0.001	1.21 (1.14, 1.28)	<0.001
Q1	226	Ref		Ref		Ref	
Q2	324	1.53 (1.29,1.81)	<0.001	1.40 (1.18, 1.66)	<0.001	1.36 (1.14, 1.61)	<0.001
Q3	388	1.89 (1.60,2.22)	<0.001	1.54 (1.31, 1.82)	<0.001	1.45 (1.23, 1.71)	<0.001
Q4	469	2.46 (2.10,2.88)	<0.001	1.91 (1.63, 2.25)	<0.001	1.74 (1.47, 2.05)	<0.001
*P*-trend		<0.001		<0.001		<0.001	
CHG-WHtR							
per 1 SD	1,407	1.38 (1.31,1.45)	<0.001	1.24 (1.17, 1.31)	<0.001	1.20 (1.13, 1.26)	<0.001
Q1	224	Ref		Ref		Ref	
Q2	308	1.46 (1.23,1.73)	<0.001	1.32 (1.11, 1.57)	0.002	1.29 (1.08, 1.53)	0.004
Q3	403	1.98 (1.68,2.34)	<0.001	1.64 (1.39, 1.94)	<0.001	1.54 (1.31, 1.83)	<0.001
Q4	472	2.49 (2.13,2.92)	<0.001	1.85 (1.57, 2.18)	<0.001	1.68 (1.42, 2.00)	<0.001
*P*-trend		<0.001		<0.001		<0.001	
CHG-WWI							
per 1 SD	1,407	1.35 (1.28,1.41)	<0.001	1.18 (1.12, 1.24)	<0.001	1.15 (1.08, 1.21)	<0.001
Q1	229	Ref		Ref		Ref	
Q2	311	1.42 (1.20,1.69)	<0.001	1.26 (1.06, 1.50)	0.008	1.23 (1.04, 1.46)	0.018
Q3	399	1.91 (1.62,2.24)	<0.001	1.51 (1.28, 1.79)	<0.001	1.43 (1.21, 1.69)	<0.001
Q4	468	2.46 (2.10,2.89)	<0.001	1.70 (1.44, 2.01)	<0.001	1.57 (1.32, 1.86)	<0.001
*P*-trend		<0.001		<0.001		<0.001	
CHG-BRI							
per 1 SD	1,407	1.32 (1.26,1.38)	<0.001	1.19 (1.13, 1.26)	<0.001	1.15 (1.09, 1.22)	<0.001
Q1	229	Ref		Ref		Ref	
Q2	318	1.47 (1.24,1.74)	<0.001	1.30 (1.10, 1.55)	0.002	1.27 (1.07, 1.50)	0.007
Q3	402	1.96 (1.67,2.30)	<0.001	1.62 (1.38, 1.91)	<0.001	1.52 (1.29, 1.79)	<0.001
Q4	458	2.31 (1.97,2.71)	<0.001	1.71 (1.45, 2.01)	<0.001	1.54 (1.30, 1.83)	<0.001
*P*-trend		<0.001		<0.001		<0.001	
CHG-ABSI							
per 1 SD	1,407	1.30 (1.24,1.37)	<0.001	1.15 (1.09, 1.21)	<0.001	1.12 (1.06, 1.18)	<0.001
Q1	242	Ref		Ref		Ref	
Q2	322	1.41 (1.19,1.66)	<0.001	1.21 (1.03, 1.44)	0.023	1.18 (1.00, 1.40)	0.050
Q3	393	1.77 (1.51,2.08)	<0.001	1.42 (1.21, 1.67)	<0.001	1.35 (1.15, 1.59)	<0.001
Q4	450	2.24 (1.92,2.62)	<0.001	1.53 (1.29, 1.80)	<0.001	1.42 (1.20, 1.68)	<0.001
*P*-trend		<0.001		<0.001		<0.001	
CHG-CVAI							
per 1 SD	1,407	1.50 (1.42,1.57)	<0.001	1.28 (1.21, 1.36)	<0.001	1.24 (1.17, 1.31)	<0.001
Q1	187	Ref		Ref		Ref	
Q2	322	1.91 (1.60,2.29)	<0.001	1.65 (1.38, 1.98)	<0.001	1.63 (1.36, 1.95)	<0.001
Q3	394	2.45 (2.06,2.92)	<0.001	1.83 (1.53, 2.19)	<0.001	1.72 (1.43, 2.06)	<0.001
Q4	504	3.50 (2.96,4.15)	<0.001	2.31 (1.93, 2.76)	<0.001	2.10 (1.74, 2.52)	<0.001
*P*-trend		<0.001		<0.001		<0.001	
TyG							
per 1 SD	1,407	1.31 (1.25,1.37)	<0.001	1.23 (1.17, 1.30)	<0.001	1.20 (1.14, 1.26)	<0.001
Q1	234	Ref		Ref		Ref	
Q2	325	1.51 (1.28,1.79)	<0.001	1.41 (1.19, 1.67)	<0.001	1.38 (1.17, 1.64)	<0.001
Q3	405	2.01 (1.71,2.36)	<0.001	1.70 (1.45, 2.00)	<0.001	1.62 (1.37, 1.91)	<0.001
Q4	443	2.33 (1.99,2.73)	<0.001	1.90 (1.62, 2.23)	<0.001	1.76 (1.50, 2.08)	<0.001
*P*-trend		<0.001		<0.001		<0.001	
AIP							
per 1 SD	1,407	1.27 (1.21,1.33)	<0.001	1.22 (1.16, 1.28)	<0.001	1.19 (1.13, 1.25)	<0.001
Q1	239	Ref		Ref		Ref	
Q2	333	1.52 (1.29,1.80)	<0.001	1.45 (1.23, 1.71)	<0.001	1.42 (1.21, 1.68)	<0.001
Q3	386	1.83 (1.56,2.15)	<0.001	1.58 (1.34, 1.86)	<0.001	1.51 (1.28, 1.78)	<0.001
Q4	449	2.25 (1.92,2.63)	<0.001	1.92 (1.64, 2.25)	<0.001	1.80 (1.53, 2.12)	<0.001
*P*-trend		<0.001		<0.001		<0.001	
METS-IR							
per 1 SD	1,407	1.31 (1.25,1.37)	<0.001	1.25 (1.19, 1.31)	<0.001	1.22 (1.15, 1.28)	<0.001
Q1	226	Ref		Ref		Ref	
Q2	320	1.47 (1.24,1.74)	<0.001	1.41 (1.19, 1.67)	<0.001	1.36 (1.15, 1.62)	<0.001
Q3	406	2.02 (1.72,2.38)	<0.001	1.75 (1.49, 2.06)	<0.001	1.64 (1.39, 1.94)	<0.001
Q4	455	2.39 (2.04,2.81)	<0.001	2.04 (1.74, 2.40)	<0.001	1.87 (1.58, 2.21)	<0.001
*P*-trend		<0.001		<0.001		<0.001	
CMI							
per 1 SD	1,407	1.08 (1.05,1.12)	<0.001	1.08 (1.04, 1.12)	<0.001	1.07 (1.03, 1.11)	0.001
Q1	235	Ref		Ref		Ref	
Q2	325	1.51 (1.28,1.78)	<0.001	1.42 (1.20, 1.68)	<0.001	1.38 (1.17, 1.64)	<0.001
Q3	404	1.97 (1.68,2.32)	<0.001	1.66 (1.41, 1.95)	<0.001	1.57(1.33, 1.85)	<0.001
Q4	443	2.25(1.92,2.64)	<0.001	1.87(1.59, 2.19)	<0.001	1.73(1.47, 2.04)	<0.001
*P*-trend		<0.001		<0.001		<0.001	

### Dose–response and threshold effect analyses

Restricted cubic spline (RCS) analyses revealed distinct dose–response patterns for the IR surrogates in Figure 2. All 12 surrogates were significantly associated with incident CVD (all *P* for overall association < 0.001), and CHG, AIP, TyG, METS-IR, CMI, CHG-CVAI, CHG-BMI, CHG-BRI, and CHG-WWI exhibited significant nonlinearity (all *P* for nonlinearity < 0.05). CHG-WC, CHG-ABSI, and CHG-WHtR did not show statistically significant nonlinear associations (all *P* for nonlinearity > 0.05). Threshold effect analyses identified a significant inflection point for all 12 surrogates, with all likelihood ratio test *P*-values < 0.05 in Supplementary Table S6. Bootstrap resampling confirmed stable inflection points for CHG, TyG, and METS-IR, with 95% percentile ranges of 5.11–5.19, 7.97–10.57, and 7.24–9.46, respectively. For the remaining nine surrogates, bootstrap ranges were wider and the inflection points were less stable; these values should therefore be regarded as exploratory rather than as precise cut-points.

**Figure 2:**
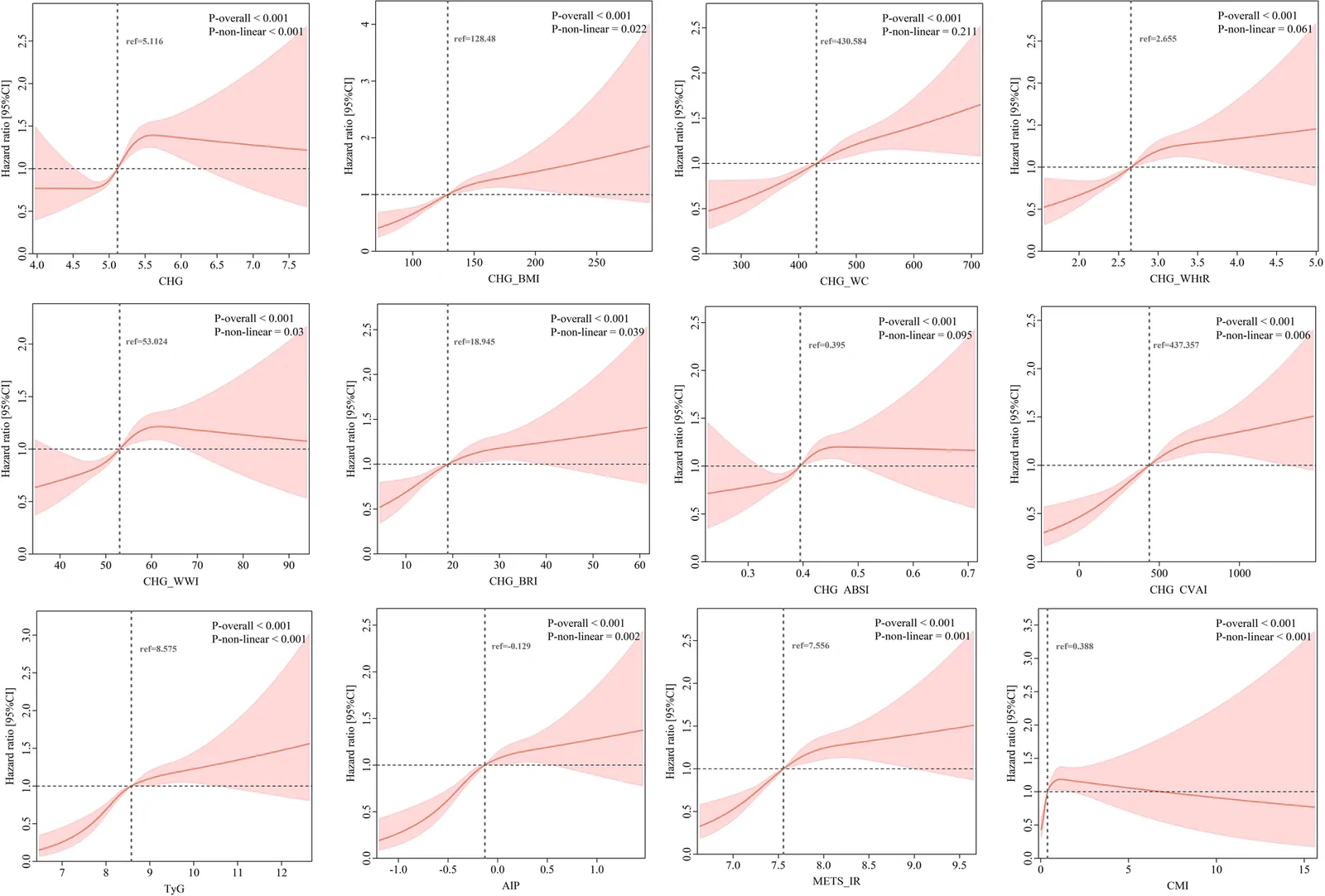
Restricted cubic spline analysis of the associations between 12 IR surrogates and CVD risk in participants with CKM syndrome stages 0–3. The model was adjusted for age, sex, residence, smoking status, drinking status, marital status, education level, hypertension, hyperuricemia, and CKD. Solid lines represent hazard ratios; shaded areas represent 95% confidence intervals; dashed vertical lines indicate reference values (median).

### Subgroup and interaction analyses

Age significantly moderated the associations of CHG-CVAI, CHG-WWI, and CHG-ABSI with CVD risk (all *P* for interaction < 0.05). In participants aged < 60 years, most IR surrogates were significantly associated with CVD risk, whereas the associations were attenuated in those aged ≥ 60 years. Residence significantly moderated the associations of CHG, CHG-WWI, CHG-ABSI, and AIP with CVD risk (all *P* for interaction < 0.05). No consistent effect modification was observed for sex, CKM syndrome stage, hypertension, or hyperuricemia. The subgroup and interaction results were attached in Supplementary Tables S7–S12.

### Sensitivity analyses

Restricting follow-up to ≥5 years, excluding participants on lipid-lowering or hypoglycemic medications, and using tertiles instead of quartiles all yielded associations consistent with the primary analysis, with HRs increasing across increasing index levels and all *P* for trend < 0.001 in Supplementary Tables S13–S15. Furthermore, the competing-risk analyses were conducted, treating non-cardiovascular death as the competing event, to assess association between all 12 IR surrogates and CVD risk, with subdistribution and age groups hazard ratios consistency of the results, confirming the robustness of our preliminary findings in Supplementary Tables S16 and S17.

### Performance of IR surrogates in predicting CVD

The C-index of the basic model was 0.667 (95% CI 0.653–0.681); after adding each IR surrogate, the C-index ranged from 0.669 to 0.676 in Table 4. In time-dependent AUC analyses at 5, 10, and 15 years, the basic model achieved AUCs of 0.792, 0.725, and 0.719, respectively; adding individual IR surrogates increased the AUC by less than 0.02 at every time point, and none of these increments was statistically significant (all *P* > 0.05) in Table 5 and Figure 3. The calibration plot showed good agreement between predicted and observed 10-year CVD-free survival in Figure 4. In NRI and IDI analyses at 10 years, adding each IR surrogate to the basic model yielded NRI values ranging from 0.089 to 0.205 (all *P* < 0.001) and IDI values ranging from 0.0004 to 0.0032 in Table 6.

**Table 4:** Discrimination of the basic model and IR surrogate-augmented models for CVD.

Model	C-index	95%CI	AUC_5Y_	95%CI	AUC_10Y_	95%CI	AUC_15Y_	95%CI
Basic model	0.667	(0.653, 0.681)	0.792	(0.745, 0.840)	0.725	(0.699, 0.751)	0.719	(0.702, 0.736)
+CHG	0.674	(0.660, 0.689)	0.805	(0.759, 0.851)	0.738	(0.712, 0.763)	0.722	(0.705, 0.739)
+CHG_BMI	0.674	(0.660, 0.688)	0.796	(0.748, 0.844)	0.735	(0.710, 0.761)	0.722	(0.705, 0.738)
+CHG_WC	0.674	(0.660, 0.688)	0.796	(0.746, 0.845)	0.738	(0.713, 0.763)	0.723	(0.706, 0.740)
+CHG_CVAI	0.676	(0.662, 0.690)	0.799	(0.751, 0.847)	0.738	(0.714, 0.763)	0.725	(0.708, 0.741)
+CHG_BRI	0.671	(0.657, 0.685)	0.796	(0.747, 0.845)	0.734	(0.709, 0.759)	0.721	(0.704, 0.738)
+CHG_WHtR	0.673	(0.659, 0.687)	0.798	(0.749, 0.847)	0.738	(0.713, 0.763)	0.722	(0.705, 0.739)
+CHG_WWI	0.671	(0.657, 0.685)	0.798	(0.750, 0.846)	0.735	(0.710, 0.760)	0.721	(0.704, 0.738)
+CHG_ABSI	0.670	(0.656, 0.684)	0.796	(0.748, 0.845)	0.733	(0.708, 0.758)	0.721	(0.704, 0.738)
+TyG	0.674	(0.660, 0.688)	0.808	(0.764, 0.852)	0.731	(0.705, 0.757)	0.724	(0.707, 0.741)
+METS_IR	0.676	(0.662, 0.690)	0.805	(0.760, 0.851)	0.734	(0.708, 0.760)	0.724	(0.708, 0.741)
+CMI	0.669	(0.655, 0.683)	0.797	(0.750, 0.843)	0.727	(0.701, 0.753)	0.721	(0.704, 0.738)
+AIP	0.674	(0.660, 0.688)	0.808	(0.764, 0.851)	0.731	(0.706, 0.757)	0.726	(0.709, 0.742)

C-index, Harrell's C; AUC, time-dependent area under the curve at 5, 10, and 15 years; values in parentheses are 95% CIs.

The basic model was adjusted for age, sex, residence, smoking status, drinking status, marital status, education level, hypertension, hyperuricemia, and CKD.

**Table 5:** Incremental discriminative value of IR surrogates over the basic model for CVD.

Model	*Δ*AUC_5Y_	*p*	*Δ*AUC_10Y_	*p*	*Δ*AUC_15Y_	*p*
Basic model	Ref		Ref		Ref	
+CHG	0.013	0.70	0.013	0.48	0.003	0.81
+CHG_BMI	0.004	0.91	0.010	0.59	0.003	0.80
+CHG_WC	0.004	0.91	0.013	0.48	0.004	0.74
+CHG_CVAI	0.007	0.84	0.013	0.48	0.006	0.62
+CHG_BRI	0.004	0.91	0.009	0.62	0.002	0.87
+CHG_WHtR	0.006	0.86	0.013	0.48	0.003	0.81
+CHG_WWI	0.006	0.86	0.010	0.59	0.002	0.87
+CHG_ABSI	0.004	0.91	0.008	0.66	0.002	0.87
+TyG	0.016	0.63	0.006	0.75	0.005	0.68
+METS_IR	0.013	0.70	0.009	0.63	0.005	0.68
+CMI	0.005	0.88	0.002	0.92	0.002	0.87
+AIP	0.016	0.63	0.006	0.75	0.007	0.56

*Δ*AUC represents the change in time-dependent AUC (5, 10, and 15 years) after adding each surrogate to the basic model; the basic model serves as the reference.

All increments were not statistically significant (all *P* > 0.05).

The basic model was adjusted for age, sex, residence, smoking status, drinking status, marital status, education level, hypertension, hyperuricemia, and CKD.

**Figure 3:**
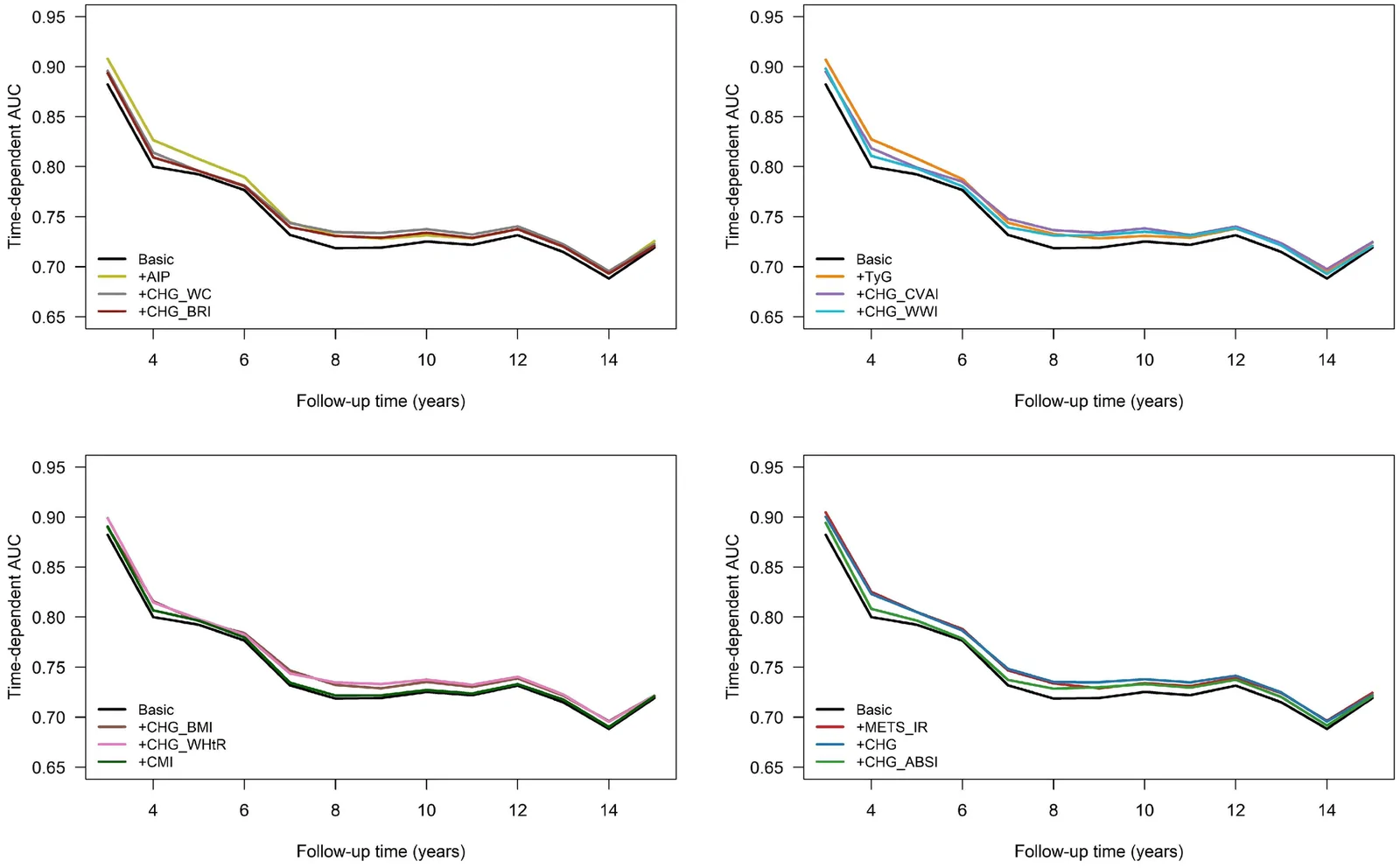
Time-dependent AUC curves for CVD of the basic model and of models additionally including each IR surrogate over the follow-up period. The basic model was adjusted for age, sex, residence, smoking status, drinking status, marital status, education level, hypertension, hyperuricemia, and CKD.

**Figure 4:**
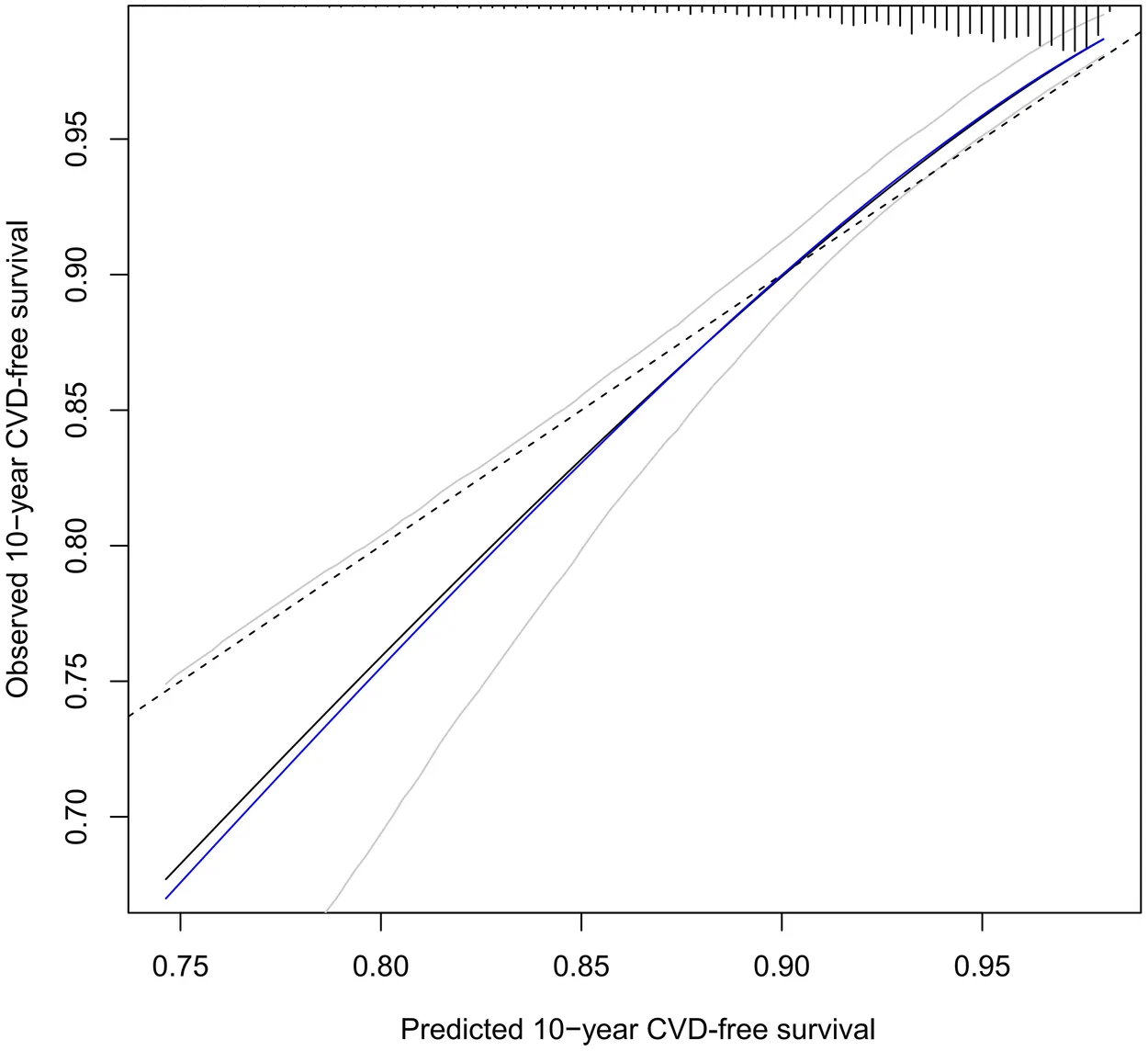
Predicted and observed 10-year CVD-free survival, based on 100 bootstrap resamples. The basic model was adjusted for age, sex, residence, smoking status, drinking status, marital status, education level, hypertension, hyperuricemia, and CKD.

**Table 6:** Improvement in 10-year risk reclassification (NRI) and integrated discrimination (IDI) with IR surrogates.

Model	NRI	95%CI	*P*	IDI	95%CI	*P*
Basic model	Ref			Ref		
+CHG	0.170	(0.112, 0.227)	<0.001	0.0027	(0.0017, 0.0037)	<0.001
+CHG_BMI	0.189	(0.131, 0.248)	<0.001	0.0032	(0.0022, 0.0043)	<0.001
+CHG_WC	0.161	(0.107, 0.229)	<0.001	0.0030	(0.0020, 0.0039)	<0.001
+CHG_CVAI	0.169	(0.110, 0.226)	<0.001	0.0032	(0.0023, 0.0041)	<0.001
+CHG_BRI	0.113	(0.052, 0.171)	<0.001	0.0018	(0.0011, 0.0025)	<0.001
+CHG_WHtR	0.147	(0.084, 0.205)	<0.001	0.0026	(0.0018, 0.0034)	<0.001
+CHG_WWI	0.137	(0.074, 0.193)	<0.001	0.0014	(0.0008, 0.0020)	<0.001
+CHG_ABSI	0.089	(0.029, 0.153)	<0.001	0.0010	(0.0004, 0.0016)	<0.001
+TyG	0.185	(0.128, 0.248)	<0.001	0.0024	(0.0016, 0.0033)	<0.001
+METS_IR	0.205	(0.146, 0.261)	<0.001	0.0030	(0.0020, 0.0039)	<0.001
+CMI	0.159	(0.105, 0.215)	<0.001	0.0004	(0.0001, 0.0007)	0.016
+AIP	0.145	(0.079, 0.200)	<0.001	0.0021	(0.0013, 0.0029)	<0.001

NRI, continuous net reclassification improvement; IDI, integrated discrimination improvement, both evaluated at 10 years.

95% CIs and *P*-values were obtained by bootstrap resampling (500 replications).

The basic model was adjusted for age, sex, residence, smoking status, drinking status, marital status, education level, hypertension, hyperuricemia, and CKD.

## Discussion

In this population-based prospective cohort of participants with stages 0–3 CKM syndrome, we systematically compared 12 insulin resistance surrogates for their association with incident CVD. All 12 surrogates were independently associated with CVD risk, with CHG-CVAI showing the strongest association. These findings indicate that simple, low-cost IR surrogates, particularly CHG-CVAI, may serve as useful complementary markers to support CVD risk stratification during the early stages of CKM syndrome.

Our study confirmed the association between IR and CVD in participants with early-stage CKM syndrome, which is consistent with previous studies conducted in the general population or participants with diabetes (4, 23). However, although a single metabolic index (e.g., the TyG index) can reflect IR, it fails to fully capture the multidimensional and interrelated pathophysiologic pathways of CKM syndrome (2). This underscores the advantage of composite indices. For example, the CHG index integrates TC, FBG, and HDL-C, thereby simultaneously assessing atherogenic lipid burden, glycemic stress, and protective lipoprotein levels; CHG-CVAI further incorporates a measure of visceral adiposity, thereby covering the core drivers of CKM syndrome—namely, dysglycemia, dyslipidemia, and central obesity—and more comprehensively reflects the clustering of multiple metabolic abnormalities already present in participants with early-stage CKM syndrome (10, 11, 24, 25). Indeed, large-scale prospective cohort studies have repeatedly demonstrated that composite indices combining surrogate markers of insulin resistance with obesity-related parameters show strong and consistent associations with CVD risk (26). Moreover, prospective cohort studies across different stages of CKM syndrome have also demonstrated that obesity-related TyG-derived indices exhibit stronger associations with CVD and mortality risk, underscoring the unique value of composite indices in early-stage CKM risk assessment (27–29).

Beyond the strength of the associations, we evaluated the predictive performance of the IR surrogates. Their discriminative ability was modest and comparable to that reported for similar composite indices combining TyG or CHG with adiposity measures in prospective studies (30, 31), likely reflecting the inherent limitation of using a single time-point metabolic marker to predict long-term CVD outcomes. Accordingly, adding these surrogates to the basic model produced only small, non-significant improvements in overall discrimination. These indices are therefore intended not to replace, but to complement, existing risk assessment scales. In this regard, they significantly improved risk reclassification beyond conventional risk factors, suggesting that they may help refine risk categorization in early-stage CKM syndrome. The limited incremental predictive value in absolute terms is consistent with the multifactorial nature of CKM syndrome. Given their complementary strengths, clinicians may select among these indices according to the specific clinical scenario, such as risk stratification or longitudinal monitoring.

The dose–response and threshold analyses further refine how IR surrogates relate to CVD risk in early-stage CKM syndrome. For most of the nonlinear indices, the association was characterized by a nonlinear pattern with a threshold, beyond which the risk increment attenuated at higher levels—in line with previous reports of threshold-type, attenuated associations for CHG- and TyG-derived indices (32, 33). Such a pattern is clinically relevant for identifying the level above which risk begins to rise. For some indices, the attenuation at very high values may partly reflect the obesity paradox, whereby the most heavily affected individuals do not necessarily show proportionally higher risk (34, 35). Together with the established role of excess adiposity as an independent risk factor for CVD (36), these findings suggest that the interpretation of a single IR surrogate should not be based on a single marker or a single cut-off, but rather on the patient's overall metabolic and clinical context.

The present study has several strengths. Its population-based prospective design enabled prospective assessment of associations between baseline IR surrogates and subsequent CVD, and its long follow-up (median 15.2 years) ensured sufficient event accrual. CVD events were ascertained through the Qingdao Cardiovascular Disease Surveillance System rather than by self-report, and the analysis focused on stages 0–3 of CKM syndrome, a critical window for CVD prevention. The study also provides a direct head-to-head comparison of 12 IR surrogates in a single prospective cohort of participants with early-stage CKM syndrome, complementing recent studies in similar populations (27–29) and offering direct comparative evidence to guide the selection of appropriate indices for specific clinical scenarios.

However, there are also some limitations in this study that should be acknowledged. First, the selection bias may influence the association due to missing main covariables or prevalent CVD at baseline. We compared the difference baseline characteristics of the included and excluded participants and no significant differences were observed between the two groups. Second, only single baseline measurements of the IR indices were used, which precluded the assessment of dynamic changes over time; future studies should incorporate repeated measurements to validate our findings. Third, because of the observational nature of this study, residual confounding could not be fully excluded despite extensive covariate adjustment. Fourth, for several indices, the data-driven inflection points had wide bootstrap confidence intervals; these threshold estimates should therefore be regarded as exploratory. Finally, the study cohort only included residents of Qingdao, China and should be caution to generalize to other population. The results required to confirm long-term prospective cohort study and other countries.

## Conclusion

In participants with stage 0–3 CKM syndrome, all IR surrogates were independently associated with CVD risk. These simple, low-cost indices may serve as complementary tools to refine CVD risk stratification and moderate performace on risk prediction in participants with early-stage CKM syndrome. A long-termand more diverse cohorts was required to validate these findings, and additionally to explore potential mechanistic pathways underlying the pathophysiology between IR surrogates and CVD risk in the future studies.

## Data Availability

The raw data supporting the conclusions of this article will be made available by the authors, without undue reservation.
