# Supplementary material for: Associations of insulin resistance surrogates with cardiovascular disease in early stage cardiovascular–kidney–metabolic syndrome: a prospective cohort study
Source: Front Cardiovasc Med. 2026 Oct 20;13:1876121. doi: 10.3389/fcvm.2026.1876121 (PMC13727414; doi:10.3389/fcvm.2026.1876121)
Supplement: Supplementary file 1 [file Datasheet1.docx]

**Table S1** Methods for Evaluating CKM Stages 0–3

| **CKM Stage** | **Detailed Criteria** |
| --- | --- |
| Stage 0  (All criteria must be met) | **Metabolic / Clinical Requirements:**  • BMI < 24 kg/m²  • WC < 80 cm (women) / < 90 cm (men)  • FBG < 100 mg/dL (5.6 mmol/L) and no diabetes  • SBP < 130 mmHg and DBP < 80 mmHg; no hypertension  • HDL-C ≥ 50 mg/dL (women) / ≥ 40 mg/dL (men)  • TG < 150 mg/dL (1.7 mmol/L)  • eGFR ≥ 60 mL/min/1.73 m²; no CKD  Exclusion:  • No subclinical or clinical CVD |
| Stage 1  Excess/dysfunctional adiposity or prediabetes  *(Must meet ≥1 metabolic criteria + ALL exclusions)* | **Metabolic / Clinical Requirements (≥1 of the following):**  • BMI ≥ 24 kg/m² (overweight/obesity)  • WC ≥ 80 cm (women) / ≥ 90 cm (men)  • Prediabetes (FBG 100–125 mg/dL)  **Other Strict Criteria (All must be met):**  • SBP < 130 mmHg and DBP < 80 mmHg; no hypertension  • TG < 150 mg/dL  • eGFR ≥ 60 mL/min/1.73 m²; no CKD  • No subclinical or clinical CVD  • FBG < 126 mg/dL; no diabetes |
| Stage 2  Metabolic risk factors or moderate CKD  *(Must meet ≥1 clinical criteria + NO CVD)* | **Clinical Criteria (≥1 of the following):**  • Hypertriglyceridemia (TG ≥ 150 mg/dL)  • Hypertension (diagnosed or BP ≥ 130/80 mmHg)  • Diabetes (diagnosed or FBG ≥ 126 mg/dL)  • Metabolic syndrome (by standard definition)  • CKD (eGFR 30–59 mL/min/1.73 m² or clinical diagnosis)  • Low HDL-C (men<40 / women<50 mg/dL)  **Exclusion:**  • No subclinical or clinical CVD |
| Stage 3  Subclinical CVD or advanced CKD  *(Must meet advanced criteria + ≥1 metabolic/clinical risk factor + NO clinical CVD)* | **Advanced Criteria (≥1 of the following):**  • eGFR < 30 mL/min/1.73 m²  • Subclinical CVD  **Metabolic/Clinical Criteria (≥1 of the following):**  • BMI ≥ 24 kg/m²  • WC ≥ 80 cm (women) / ≥ 90 cm (men)  • Prediabetes  • Hypertriglyceridemia  • Hypertension  • Diabetes  • Metabolic syndrome  • CKD (eGFR 30–59 mL/min/1.73 m²)  **Exclusion:**  • No clinical CVD |

| **Table S2** Formulas for IR surrogates and Adiposity Indices | | | |
| --- | --- | --- | --- |
| **Variable** | **Formula** | **Description** | **Units note** |
| BRI | BRI = 364.2 - 365.5 × √[1 - (WC/(2π))² / (0.5 × Height)²] | Body Roundness Index | WC and Height in cm |
| WWI | WWI = WC / √Weight | weight-adjusted waist index | WC in cm, Weight in kg |
| ABSI | ABSI = WC / [BMI^(2/3) × Height^(1/2)] | A Body Shape Index | WC and Height converted to meters |
| CVAI | Male: CVAI = -267.93 + 0.68 × Age + 0.03 × BMI + 4.00 × WC + 22.00 × log₁₀(TG) - 16.32 × HDL-C  Female: CVAI = -187.32 + 1.71 × Age + 4.23 × BMI + 1.12 × WC + 39.76 × log₁₀(TG) - 11.66 × HDL-C | Chinese Visceral Adiposity Index | Age in years, BMI in kg/m², WC in cm, TG and HDL-C converted to mmol/L. Note: TG_mmol/L = TG/88.57, HDL-C_mmol/L = HDL-C/38.67 |
| CHG | CHG=ln[TC×FBG/(2×HDL-C)] | Cholesterol-HDL-Glucose  Index | TC, HDL-C and FBG in mg/dL |
| CHG-BMI | CHG-BMI = CHG × BMI | CHG index multiplied by BMI | CHG unitless, BMI in kg/m² |
| CHG-WC | CHG-WC = CHG × Waist Circumference | CHG index multiplied by Waist Circumference | CHG unitless, WC in cm |
| CHG-WHtR | CHG-WHtR = CHG× (WC / Height) | CHG index multiplied by Waist-to-Height Ratio | CHG unitless, WC and Height in cm |
| CHG-BRI | CHG-BRI = CHG × BRI | CHG index multiplied by BRI | CHG unitless, BRI unitless |
| CHG-ABSI | CHG-ABSI = CHG × ABSI | CHG index multiplied by ABSI | CHG unitless, ABSI unitless |
| CHG-WWI | CHG-WWI = CHG× WWI | CHG index multiplied by WWI | CHG unitless, WWI unitless |
| CHG-CVAI | CHG-CVAI = CHG× CVAI | CHG index multiplied by CVAI | CHG unitless, CVAI unitless |
| METS-IR | METS-IR = ln[(2 × FBG + TG) × BMI / ln(HDL-C)] | Metabolic score for Insulin Resistance | FBG and TG in mg/dL, BMI in kg/m², HDL-C in mg/dL |
| TyG | TyG = ln[TG × FBG/2] | Triglyceride-glucose index | TG and Glucose in mg/dL |
| AIP | AIP = log₁₀(TG/HDL-C) | Atherogenic Index of Plasma | TG and HDL-C in mmol/L |
| CMI | CMI=TG/HDL-C× (WC / Height) | Cardiac Metabolic Index | TG and HDL-C in mmol/L,WC and Height in cm |

**Table S3** Variance inflation factors (VIF) for the 12 IR surrogates and covariates in single-index Model 3

| **IR surrogates** | **VIF of the surrogate** |
| --- | --- |
| CHG | 1.09 |
| CHG-BMI | 1.13 |
| CHG-WC | 1.17 |
| CHG-CVAI | 1.33 |
| CHG-BRI | 1.19 |
| CHG-WHtR | 1.20 |
| CHG-WWI | 1.21 |
| CHG-ABSI | 1.16 |
| TyG | 1.11 |
| METS-IR | 1.13 |
| CMI | 1.04 |
| AIP | 1.09 |
| Each single-index Model 3 included the indicated IR surrogate plus 10 covariates. The GVIF^(1/(2·Df)) values of the covariates were as follows: age, 1.19; sex, 1.58; education level, 1.09; smoking status, 1.20; drinking status, 1.18; residence, 1.20; marital status, 1.02; hypertension, 1.06; hyperuricemia, 1.02; and CKD, 1.00 (all < 5). The GVIF^(1/(2·Df)) values of the 12 IR surrogates ranged from 1.04 to 1.33 (all < 5). These results indicate no serious multicollinearity in any single-index model. | |

**Table S4** Benjamini–Hochberg false discovery rate correction for *P* for trend of 12 IR surrogates (Model 3)

| **IR surrogates** | ***P* for trend** | **FDR q** |
| --- | --- | --- |
| CHG | <0.001 | 0.001 |
| CHG-BMI | <0.001 | 0.001 |
| CHG-WC | <0.001 | 0.001 |
| CHG-CVAI | <0.001 | 0.001 |
| CHG-BRI | <0.001 | 0.001 |
| CHG-WHtR | <0.001 | 0.001 |
| CHG-WWI | <0.001 | 0.001 |
| CHG-ABSI | <0.001 | 0.001 |
| TyG | <0.001 | 0.001 |
| METS-IR | <0.001 | 0.001 |
| AIP | <0.001 | 0.001 |
| CMI | <0.001 | 0.001 |
| *P* for trend was derived from Model 3 (adjusted for age, sex, residence, smoking status, drinking status, marital status, education level, hypertension, hyperuricemia, and CKD), treating the quartile variable as a continuous variable. FDR q values were calculated using the Benjamini–Hochberg procedure. Because all 12 indices were significant at *P* for trend < 0.001, the FDR-adjusted q values were all < 0.001, indicating that the associations remained significant after correction for multiple testing.  *P* for trend values < 0.001 were recorded as 0.001 before applying the BH correction; the resulting q values are therefore conservative upper bounds.  FDR, false discovery rate; Model 3 as defined in Table 3. | | |

**Table S5** Proportional-hazards assumption and sensitivity analyses for the 12 IR surrogates (Model 3)

| **IR surrogates** | **Schoenfeld test *P* (Q4)** | **Time interaction *P*** | **>8-y follow-up Q4 HR (95% CI)** | **PH assumption** |
| --- | --- | --- | --- | --- |
| CHG | 0.310 | 0.020 | 1.69 (1.42, 2.02) | Satisfied |
| CHG-BMI | 0.602 | 0.066 | 1.77 (1.48, 2.11) | Satisfied |
| CHG-WC | 0.387 | 0.044 | 1.70 (1.42, 2.03) | Satisfied |
| CHG-CVAI | 0.024 | 0.002 | 1.94 (1.60, 2.36) | Violated |
| CHG-BRI | 0.108 | 0.021 | 1.45 (1.21, 1.73) | Satisfied |
| CHG-WHtR | 0.046 | 0.004 | 1.55 (1.30, 1.86) | Violated |
| CHG-WWI | 0.005 | 0.002 | 1.43 (1.19, 1.71) | Violated |
| CHG-ABSI | 0.218 | 0.010 | 1.36 (1.14, 1.63) | Satisfied |
| TyG | 0.236 | 0.143 | 1.63 (1.37, 1.95) | Satisfied |
| METS-IR | 0.570 | 0.118 | 1.79 (1.50, 2.14) | Satisfied |
| AIP | 0.172 | 0.080 | 1.67 (1.41, 1.99) | Satisfied |
| CMI | 0.112 | 0.028 | 1.60 (1.35, 1.91) | Satisfied |
| The proportional-hazards (PH) assumption was tested using Schoenfeld residuals (*P* for the highest quartile of each index); *P* < 0.05 indicated violation of the PH assumption, otherwise the assumption was considered satisfied. Because of the long follow-up, the global PH test was significant for several models, which was largely attributable to the residence covariate. Time-interaction models (index × ln(time)) and analyses stratified by follow-up duration (≤8 vs. >8 years) were fitted as sensitivity analyses. The PH-assumption classification was based a priori on the Schoenfeld test for the highest quartile. Three indices (CHG-CVAI, CHG-WHtR, and CHG-WWI) showed evidence of PH violation. For five indices (CHG, CHG-WC, CHG-BRI, CHG-ABSI, and CMI), the time-interaction term was nominally significant (*P* < 0.05) despite a non-significant Schoenfeld test; because these indices exhibited associations of consistent direction and significance in the >8-year stratum, they were classified as satisfying the PH assumption, and the time-interaction *P* values are reported for transparency. All indices, including the three with PH violation, showed associations of consistent direction and significance in the >8-year follow-up stratum, indicating that PH violations did not materially affect the conclusions. The ≤8-year stratum (n=400, 203 events) had limited statistical power. | | | | |

**Table S6** Data-derived inflection points of 12 IR surrogates identified by two-piecewise linear regression, with bootstrap 95% percentile ranges

| **IR surrogates** | **Inflection point** | **Bootstrap 95% ranges** | ***P* for LRT** |
| --- | --- | --- | --- |
| CHG | 5.155 | (5.111, 5.185) | <0.001 |
| CHG-BMI | 116.560 | (105.394, 190.774) | 0.002 |
| CHG-WC | 419.887 | (357.714, 710.425) | 0.010 |
| CHG-CVAI | 323.521 | (214.521, 442.303) | <0.001 |
| CHG-BRI | 13.114 | (11.426, 20.932) | 0.027 |
| CHG-WHtR | 2.637 | (2.226, 4.959) | 0.007 |
| CHG-WWI | 53.172 | (46.593, 86.148) | 0.003 |
| CHG-ABSI | 0.394 | (0.345, 0.707) | 0.010 |
| TyG | 8.034 | (7.972, 10.569) | <0.001 |
| METS-IR | 7.337 | (7.242, 9.464) | <0.001 |
| CMI | 0.187 | (0.047, 0.210) | <0.001 |
| AIP | −0.388 | (−0.442, 1.251) | <0.001 |
| Abbreviations: LRT, likelihood ratio test.  Inflection points were identified using two-piecewise linear regression for each IR surrogate; *P* for LRT compares the two-piecewise linear model with a standard linear model. The bootstrap 95% percentile ranges were obtained by extracting the 2.5th and 97.5th percentiles of inflection-point distribution across 1000 bootstrap resamples. For all surrogates except CHG, TyG, and METS-IR (i.e., CHG-BMI, CHG-WC, CHG-CVAI, CHG-BRI, CHG-WHtR, CHG-WWI, CHG-ABSI, CMI, and AIP), the bootstrap 95% percentile ranges were relatively wide; the thresholds for these indices were therefore considered exploratory, and the nonlinear association was primarily interpreted rather than the exact cut-point. | | | |

| **Table S7** Association between various IR surrogates and CVD incidence in individuals with CKM syndrome stages 0-3 by age | | | | | |
| --- | --- | --- | --- | --- | --- |
| **IR surrogates**  **(Quartile)** | **Age<60** | | **Age>=60** | | ***P* for interaction** |
|  | **HR (95% CI)** | ***P*** | **HR (95% CI)** | ***P*** |  |
| CHG | | | | | |
| Q1 | Ref |  | Ref |  | 0.258 |
| Q2 | 1.38 (1.13, 1.69) | **0.002** | 0.95 (0.68, 1.33) | 0.775 |  |
| Q3 | 1.81 (1.48, 2.20) | **<0.001** | 1.45 (1.07, 1.96) | **0.018** |  |
| Q4 | 1.97 (1.62, 2.41) | **<0.001** | 1.52 (1.13, 2.06) | **0.006** |  |
| CHG-BMI | | | | | |
| Q1 | Ref |  | Ref |  | 0.727 |
| Q2 | 1.33 (1.08, 1.63) | **0.007** | 1.24 (0.92, 1.67) | 0.157 |  |
| Q3 | 1.42 (1.15, 1.74) | **0.001** | 1.38 (1.03, 1.85) | **0.029** |  |
| Q4 | 1.80 (1.47, 2.20) | **<0.001** | 1.64 (1.23, 2.19) | **0.001** |  |
| CHG-WC | | | | | |
| Q1 | Ref |  | Ref |  | 0.310 |
| Q2 | 1.39 (1.14, 1.70) | **0.001** | 1.26 (0.91, 1.76) | 0.161 |  |
| Q3 | 1.59 (1.30, 1.94) | **<0.001** | 1.27 (0.93, 1.74) | 0.139 |  |
| Q4 | 1.93 (1.57, 2.36) | **<0.001** | 1.48 (1.09, 2.02) | **0.012** |  |
| CHG-CVAI | | | | | |
| Q1 | Ref |  | Ref |  | **0.023** |
| Q2 | 1.68 (1.38, 2.06) | **<0.001** | 1.59 (1.04, 2.43) | **0.032** |  |
| Q3 | 2.08 (1.70, 2.55) | **<0.001** | 1.30 (0.86, 1.97) | 0.215 |  |
| Q4 | 2.50 (2.02, 3.09) | **<0.001** | 1.85 (1.24, 2.76) | **0.002** |  |
| CHG-BRI | | | | | |
| Q1 | Ref |  | Ref |  | 0.191 |
| Q2 | 1.26 (1.03, 1.53) | **0.025** | 1.32 (0.94, 1.85) | 0.115 |  |
| Q3 | 1.61 (1.33, 1.95) | **<0.001** | 1.45 (1.04, 2.01) | **0.029** |  |
| Q4 | 1.71 (1.40, 2.10) | **<0.001** | 1.44 (1.04, 1.98) | **0.028** |  |
| CHG-WHtR | | | | | |
| Q1 | Ref |  | Ref |  | 0.170 |
| Q2 | 1.31 (1.07, 1.60) | **0.009** | 1.27 (0.90, 1.81) | 0.174 |  |
| Q3 | 1.64 (1.35, 1.99) | **<0.001** | 1.45 (1.04, 2.02) | **0.028** |  |
| Q4 | 1.91 (1.56, 2.34) | **<0.001** | 1.52 (1.10, 2.10) | **0.011** |  |
| CHG-WWI | | | | | |
| Q1 | Ref |  | Ref |  | 0.023 |
| Q2 | 1.24 (1.02, 1.51) | 0.030 | 1.07 (0.75, 1.53) | 0.699 |  |
| Q3 | 1.49 (1.23, 1.81) | <0.001 | 1.09 (0.78, 1.53) | 0.603 |  |
| Q4 | 1.74 (1.42, 2.12) | <0.001 | 1.16 (0.84, 1.60) | 0.373 |  |
| CHG-ABSI | | | | | |
| Q1 | Ref |  | Ref |  | **0.003** |
| Q2 | 1.23 (1.01, 1.49) | **0.037** | 1.10 (0.78, 1.55) | 0.599 |  |
| Q3 | 1.61 (1.33, 1.94) | **<0.001** | 1.00 (0.71, 1.40) | 0.982 |  |
| Q4 | 1.81 (1.49, 2.21) | **<0.001** | 1.10 (0.80, 1.52) | 0.547 |  |
| TyG | | | | | |
| Q1 | Ref |  | Ref |  | 0.131 |
| Q2 | 1.43 (1.17, 1.75) | **<0.001** | 1.33 (0.98, 1.82) | 0.069 |  |
| Q3 | 1.75 (1.44, 2.13) | **<0.001** | 1.44 (1.07, 1.94) | **0.015** |  |
| Q4 | 2.03 (1.67, 2.48) | **<0.001** | 1.48 (1.09, 2.00) | **0.011** |  |
| CMI | | | | | |
| Q1 | Ref |  | Ref |  | 0.097 |
| Q2 | 1.43 (1.16, 1.75) | **0.001** | 1.28 (0.94, 1.73) | 0.116 |  |
| Q3 | 1.76 (1.44, 2.14) | **<0.001** | 1.31 (0.98, 1.76) | 0.067 |  |
| Q4 | 1.93 (1.58, 2.37) | **<0.001** | 1.44 (1.07, 1.94) | **0.016** |  |
| METS-IR | | | | | |
| Q1 | Ref |  | Ref |  | 0.104 |
| Q2 | 1.47 (1.19, 1.81) | **<0.001** | 1.15 (0.85, 1.56) | 0.375 |  |
| Q3 | 1.83 (1.49, 2.24) | **<0.001** | 1.32 (0.99, 1.77) | 0.060 |  |
| Q4 | 2.10 (1.71, 2.57) | **<0.001** | 1.49 (1.11, 2.00) | **0.008** |  |
| AIP | | | | | |
| Q1 | Ref |  | Ref |  | 0.147 |
| Q2 | 1.46 (1.20, 1.79) | **<0.001** | 1.34 (1.00, 1.81) | 0.050 |  |
| Q3 | 1.69 (1.38, 2.06) | **<0.001** | 1.30 (0.97, 1.73) | 0.077 |  |
| Q4 | 1.98 (1.62, 2.41) | **<0.001** | 1.54 (1.15, 2.05) | **0.004** |  |
| Q1: Quartile 1, Q2: Quartile 2, Q3: Quartile 3, Q4: Quartile 4.  Adjusted for sex, residence, smoking status, drinking status, marital status, education level, hypertension, hyperuricemia, and CKD (age excluded because it is the stratifying variable). | | | | | |

| **Table S8** Association between various IR surrogates and CVD incidence in individuals with CKM syndrome stages 0-3 by residence | | | | | |
| --- | --- | --- | --- | --- | --- |
| **IR surrogates**  **(Quartile)** | **Urban** | | **Rural** | | ***P* for interaction** |
|  | **HR (95% CI)** | ***P*** | **HR (95% CI)** | ***P*** |  |
| CHG | | | | | |
| Q1 | Ref |  | Ref |  | **0.043** |
| Q2 | 1.44 (1.03, 2.01) | **0.033** | 1.14 (0.93, 1.40) | 0.206 |  |
| Q3 | 2.29 (1.68, 3.13) | **<0.001** | 1.41 (1.16, 1.72) | **0.001** |  |
| Q4 | 2.22 (1.62, 3.04) | **<0.001** | 1.57 (1.29, 1.91) | **<0.001** |  |
| CHG-BMI | | | | | |
| Q1 | Ref |  | Ref |  | 0.623 |
| Q2 | 1.41 (1.02, 1.94) | **0.038** | 1.27 (1.04, 1.55) | **0.019** |  |
| Q3 | 1.50 (1.09, 2.07) | **0.013** | 1.37 (1.12, 1.67) | **0.002** |  |
| Q4 | 2.15 (1.58, 2.94) | **<0.001** | 1.61 (1.32, 1.96) | **<0.001** |  |
| CHG-WC | | | | | |
| Q1 | Ref |  | Ref |  | 0.275 |
| Q2 | 1.43 (1.02, 2.02) | **0.040** | 1.34 (1.10, 1.63) | **0.004** |  |
| Q3 | 1.60 (1.14, 2.23) | **0.006** | 1.37 (1.13, 1.67) | **0.002** |  |
| Q4 | 2.16 (1.54, 3.03) | **<0.001** | 1.54 (1.26, 1.88) | **<0.001** |  |
| CHG-CVAI | | | | | |
| Q1 | Ref |  | Ref |  | 0.128 |
| Q2 | 1.89 (1.27, 2.81) | **0.002** | 1.55 (1.26, 1.91) | **<0.001** |  |
| Q3 | 2.06 (1.38, 3.07) | **<0.001** | 1.60 (1.30, 1.97) | **<0.001** |  |
| Q4 | 2.93 (1.96, 4.39) | **<0.001** | 1.80 (1.45, 2.23) | **<0.001** |  |
| CHG-BRI | | | | | |
| Q1 | Ref |  | Ref |  | 0.196 |
| Q2 | 1.39 (1.00, 1.94) | 0.050 | 1.21 (0.99, 1.48) | 0.067 |  |
| Q3 | 1.82 (1.32, 2.51) | **<0.001** | 1.38 (1.13, 1.68) | **0.002** |  |
| Q4 | 1.99 (1.43, 2.77) | **<0.001** | 1.33 (1.08, 1.62) | **0.006** |  |
| CHG-WHtR | | | | | |
| Q1 | Ref |  | Ref |  | 0.160 |
| Q2 | 1.25 (0.90, 1.75) | 0.185 | 1.29 (1.05, 1.58) | **0.015** |  |
| Q3 | 1.76 (1.28, 2.42) | **<0.001** | 1.43 (1.17, 1.75) | **<0.001** |  |
| Q4 | 2.08 (1.50, 2.88) | **<0.001** | 1.47 (1.20, 1.80) | **<0.001** |  |
| CHG-WWI | | | | | |
| Q1 | Ref |  | Ref |  | 0.011 |
| Q2 | 1.70 (1.20, 2.39) | 0.002 | 1.09 (0.89, 1.33) | 0.413 |  |
| Q3 | 2.06 (1.48, 2.87) | <0.001 | 1.22 (1.00, 1.48) | 0.051 |  |
| Q4 | 2.43 (1.72, 3.42) | <0.001 | 1.29 (1.05, 1.57) | 0.014 |  |
| CHG-ABSI | | | | | |
| Q1 | Ref |  | Ref |  | **0.044** |
| Q2 | 1.52 (1.09, 2.12) | **0.014** | 1.08 (0.89, 1.32) | 0.428 |  |
| Q3 | 1.78 (1.29, 2.47) | **0.001** | 1.20 (0.99, 1.45) | 0.069 |  |
| Q4 | 1.92 (1.37, 2.68) | **<0.001** | 1.23 (1.01, 1.50) | **0.039** |  |
| TyG | | | | | |
| Q1 | Ref |  | Ref |  | 0.056 |
| Q2 | 1.96 (1.39, 2.77) | **<0.001** | 1.24 (1.02, 1.51) | **0.031** |  |
| Q3 | 2.35 (1.69, 3.26) | **<0.001** | 1.38 (1.13, 1.67) | **0.001** |  |
| Q4 | 2.50 (1.80, 3.48) | **<0.001** | 1.54 (1.27, 1.87) | **<0.001** |  |
| CMI | | | | | |
| Q1 | Ref |  | Ref |  | 0.056 |
| Q2 | 1.99 (1.38, 2.86) | **<0.001** | 1.24 (1.02, 1.50) | **0.031** |  |
| Q3 | 2.27 (1.59, 3.22) | **<0.001** | 1.37 (1.13, 1.67) | **0.001** |  |
| Q4 | 2.61 (1.83, 3.72) | **<0.001** | 1.48 (1.21, 1.79) | **<0.001** |  |
| METS-IR | | | | | |
| Q1 | Ref |  | Ref |  | 0.372 |
| Q2 | 1.52 (1.08, 2.15) | **0.016** | 1.28 (1.05, 1.56) | **0.016** |  |
| Q3 | 1.95 (1.41, 2.70) | **<0.001** | 1.50 (1.23, 1.83) | **<0.001** |  |
| Q4 | 2.43 (1.76, 3.37) | **<0.001** | 1.62 (1.33, 1.98) | **<0.001** |  |
| AIP | | | | | |
| Q1 | Ref |  | Ref |  | **0.005** |
| Q2 | 2.40 (1.67, 3.44) | **<0.001** | 1.19 (0.98, 1.45) | 0.074 |  |
| Q3 | 2.40 (1.68, 3.41) | **<0.001** | 1.28 (1.06, 1.55) | **0.011** |  |
| Q4 | 2.79 (1.96, 3.97) | **<0.001** | 1.56 (1.29, 1.88) | **<0.001** |  |
| Q1: Quartile 1, Q2: Quartile 2, Q3: Quartile 3, Q4: Quartile 4.  Adjusted for age, sex, smoking status, drinking status, marital status, education level, hypertension, hyperuricemia, and CKD (residence excluded because it is the stratifying variable). | | | | | |

| **Table S9** Association between various IR surrogates and CVD incidence in individuals with CKM syndrome stages 0-3 by sex | | | | | |
| --- | --- | --- | --- | --- | --- |
| **IR surrogates**  **(Quartile)** | **Men** | | **Women** | | ***P* for interaction** |
|  | **HR (95% CI)** | ***P*** | **HR (95% CI)** | ***P*** |  |
| CHG | | | | | |
| Q1 | Ref |  | Ref |  | 0.678 |
| Q2 | 1.16 (0.86, 1.55) | 0.332 | 1.25 (1.00, 1.55) | **0.045** |  |
| Q3 | 1.52 (1.15, 2.01) | **0.003** | 1.74 (1.42, 2.14) | **<0.001** |  |
| Q4 | 1.80 (1.37, 2.37) | **<0.001** | 1.72 (1.39, 2.12) | **<0.001** |  |
| CHG- BMI | | | | | |
| Q1 | Ref |  | Ref |  | 0.842 |
| Q2 | 1.38 (1.05, 1.81) | **0.020** | 1.27 (1.02, 1.58) | **0.030** |  |
| Q3 | 1.57 (1.21, 2.05) | **0.001** | 1.31 (1.06, 1.63) | **0.014** |  |
| Q4 | 1.96 (1.49, 2.56) | **<0.001** | 1.69 (1.37, 2.09) | **<0.001** |  |
| CHG- WC | | | | | |
| Q1 | Ref |  | Ref |  | 0.205 |
| Q2 | 1.60 (1.19, 2.16) | **0.002** | 1.23 (1.00, 1.52) | **0.049** |  |
| Q3 | 1.51 (1.11, 2.04) | **0.008** | 1.39 (1.13, 1.71) | **0.002** |  |
| Q4 | 2.17 (1.63, 2.89) | **<0.001** | 1.51 (1.22, 1.87) | **<0.001** |  |
| CHG- CVAI | | | | | |
| Q1 | Ref |  | Ref |  | 0.376 |
| Q2 | 1.71 (1.29, 2.28) | **<0.001** | 1.59 (1.25, 2.02) | **<0.001** |  |
| Q3 | 1.56 (1.17, 2.08) | **0.003** | 1.83 (1.44, 2.34) | **<0.001** |  |
| Q4 | 2.20 (1.67, 2.90) | **<0.001** | 2.12 (1.63, 2.77) | **<0.001** |  |
| CHG- BRI | | | | | |
| Q1 | Ref |  | Ref |  | 0.487 |
| Q2 | 1.25 (0.95, 1.63) | 0.106 | 1.24 (0.99, 1.56) | 0.060 |  |
| Q3 | 1.58 (1.22, 2.05) | **0.001** | 1.44 (1.16, 1.80) | **0.001** |  |
| Q4 | 1.83 (1.40, 2.40) | **<0.001** | 1.36 (1.09, 1.69) | **0.007** |  |
| CHG- WHtR | | | | | |
| Q1 | Ref |  | Ref |  | 0.623 |
| Q2 | 1.32 (1.01, 1.74) | **0.043** | 1.24 (0.99, 1.56) | 0.061 |  |
| Q3 | 1.58 (1.21, 2.07) | **0.001** | 1.48 (1.19, 1.85) | **<0.001** |  |
| Q4 | 1.97 (1.50, 2.59) | **<0.001** | 1.50 (1.20, 1.87) | **<0.001** |  |
| CHG- WWI | | | | | |
| Q1 | Ref |  | Ref |  | 0.171 |
| Q2 | 1.31 (0.99, 1.72) | 0.060 | 1.17 (0.94, 1.46) | 0.159 |  |
| Q3 | 1.41 (1.07, 1.84) | 0.014 | 1.40 (1.13, 1.74) | 0.002 |  |
| Q4 | 1.91 (1.46, 2.51) | <0.001 | 1.36 (1.09, 1.70) | 0.007 |  |
| CHG- ABSI | | | | | |
| Q1 | Ref |  | Ref |  | 0.166 |
| Q2 | 1.26 (0.93, 1.71) | 0.132 | 1.12 (0.91, 1.37) | 0.278 |  |
| Q3 | 1.35 (1.01, 1.81) | **0.044** | 1.33 (1.08, 1.63) | **0.006** |  |
| Q4 | 1.72 (1.29, 2.29) | **<0.001** | 1.23 (0.99, 1.53) | 0.057 |  |
| TyG | | | | | |
| Q1 | Ref |  | Ref |  | 0.640 |
| Q2 | 1.37 (1.05, 1.80) | **0.021** | 1.41 (1.13, 1.75) | **0.002** |  |
| Q3 | 1.63 (1.26, 2.12) | **<0.001** | 1.61 (1.30, 2.00) | **<0.001** |  |
| Q4 | 1.60 (1.23, 2.08) | **0.001** | 1.86 (1.49, 2.31) | **<0.001** |  |
| CMI | | | | | |
| Q1 | Ref |  | Ref |  | 0.850 |
| Q2 | 1.34 (1.02, 1.75) | **0.034** | 1.42 (1.14, 1.76) | **0.002** |  |
| Q3 | 1.65 (1.27, 2.14) | **<0.001** | 1.54 (1.24, 1.90) | **<0.001** |  |
| Q4 | 1.68 (1.28, 2.20) | **<0.001** | 1.74 (1.40, 2.16) | **<0.001** |  |
| METS-IR | | | | | |
| Q1 | Ref |  | Ref |  | 0.890 |
| Q2 | 1.29 (0.98, 1.69) | 0.071 | 1.41 (1.13, 1.77) | **0.002** |  |
| Q3 | 1.56 (1.19, 2.05) | **0.001** | 1.70 (1.37, 2.11) | **<0.001** |  |
| Q4 | 1.79 (1.36, 2.34) | **<0.001** | 1.91 (1.53, 2.38) | **<0.001** |  |
| AIP | | | | | |
| Q1 | Ref |  | Ref |  | 0.875 |
| Q2 | 1.43 (1.09, 1.87) | **0.009** | 1.42 (1.15, 1.76) | **0.001** |  |
| Q3 | 1.57 (1.20, 2.06) | **0.001** | 1.49 (1.21, 1.84) | **<0.001** |  |
| Q4 | 1.72 (1.31, 2.26) | **<0.001** | 1.82 (1.47, 2.24) | **<0.001** |  |
| Q1: Quartile 1, Q2: Quartile 2, Q3: Quartile 3, Q4: Quartile 4.  Adjusted for age, residence, smoking status, drinking status, marital status, education level, hypertension, hyperuricemia, and CKD (sex excluded because it is the stratifying variable). | | | | | |

| **Table S10** Association between various IR surrogates and CVD incidence in individuals by CKM syndrome stages | | | | | |
| --- | --- | --- | --- | --- | --- |
| **IR surrogates**  **(Quartile)** | **Stage0-1** | | **Stage2-3** | | ***P* for interaction** |
|  | **HR(95% CI)** | ***P*** | **HR(95% CI)** | ***P*** |  |
| CHG | | | | | |
| Q1 | Ref |  | Ref |  | 0.194 |
| Q2 | 1.15 (0.80, 1.64) | 0.452 | 1.25 (1.02, 1.52) | 0.033 |  |
| Q3 | 1.02 (0.66, 1.59) | 0.930 | 1.77 (1.47, 2.13) | <0.001 |  |
| Q4 | 1.58 (0.87, 2.86) | 0.130 | 1.81 (1.50, 2.17) | <0.001 |  |
| CHG- BMI | | | | | |
| Q1 | Ref |  | Ref |  | 0.624 |
| Q2 | 1.42 (1.00, 2.01) | 0.051 | 1.29 (1.06, 1.57) | 0.010 |  |
| Q3 | 1.47 (0.95, 2.28) | 0.082 | 1.39 (1.15, 1.68) | 0.001 |  |
| Q4 | 1.26 (0.64, 2.47) | 0.500 | 1.79 (1.49, 2.15) | <0.001 |  |
| CHG- WC | | | | | |
| Q1 | Ref |  | Ref |  | 0.439 |
| Q2 | 1.17 (0.83, 1.64) | 0.380 | 1.43 (1.17, 1.75) | <0.001 |  |
| Q3 | 1.02 (0.64, 1.63) | 0.926 | 1.51 (1.25, 1.84) | <0.001 |  |
| Q4 | 1.76 (0.94, 3.30) | 0.079 | 1.78 (1.48, 2.15) | <0.001 |  |
| CHG- CVAI | | | | | |
| Q1 | Ref |  | Ref |  | 0.589 |
| Q2 | 1.46 (1.03, 2.09) | 0.035 | 1.68 (1.35, 2.09) | <0.001 |  |
| Q3 | 1.68 (1.07, 2.66) | 0.025 | 1.73 (1.40, 2.15) | <0.001 |  |
| Q4 | 1.21 (0.51, 2.87) | 0.658 | 2.15 (1.74, 2.66) | <0.001 |  |
| CHG- BRI | | | | | |
| Q1 | Ref |  | Ref |  | 0.798 |
| Q2 | 1.19 (0.83, 1.71) | 0.337 | 1.28 (1.05, 1.57) | 0.013 |  |
| Q3 | 1.26 (0.82, 1.95) | 0.294 | 1.54 (1.28, 1.86) | <0.001 |  |
| Q4 | 1.36 (0.75, 2.47) | 0.316 | 1.55 (1.29, 1.87) | <0.001 |  |
| CHG- WHtR | | | | | |
| Q1 | Ref |  | Ref |  | 0.832 |
| Q2 | 1.34 (0.94, 1.90) | 0.103 | 1.26 (1.03, 1.55) | 0.022 |  |
| Q3 | 1.35 (0.87, 2.10) | 0.181 | 1.54 (1.27, 1.86) | <0.001 |  |
| Q4 | 1.42 (0.72, 2.81) | 0.312 | 1.67 (1.38, 2.01) | <0.001 |  |
| CHG- WWI | | | | | |
| Q1 | Ref |  | Ref |  | 0.315 |
| Q2 | 0.96 (0.67, 1.38) | 0.825 | 1.32 (1.08, 1.61) | 0.006 |  |
| Q3 | 1.03 (0.65, 1.64) | 0.887 | 1.51 (1.25, 1.82) | <0.001 |  |
| Q4 | 1.00 (0.57, 1.75) | 0.993 | 1.66 (1.37, 2.01) | <0.001 |  |
| CHG- ABSI | | | | | |
| Q1 | Ref |  | Ref |  | 0.417 |
| Q2 | 1.03 (0.72, 1.48) | 0.861 | 1.22 (1.01, 1.48) | 0.041 |  |
| Q3 | 0.93 (0.59, 1.46) | 0.741 | 1.42 (1.18, 1.70) | <0.001 |  |
| Q4 | 1.18 (0.71, 1.97) | 0.531 | 1.46 (1.22, 1.76) | <0.001 |  |
| TyG | | | | | |
| Q1 | Ref |  | Ref |  | 0.587 |
| Q2 | 1.35 (0.96, 1.89) | 0.083 | 1.41 (1.16, 1.72) | 0.001 |  |
| Q3 | 1.38 (0.90, 2.11) | 0.141 | 1.66 (1.38, 2.00) | <0.001 |  |
| Q4 | - |  | 1.78 (1.48, 2.13) | <0.001 |  |
| CMI | | | | | |
| Q1 | Ref |  | Ref |  | 0.171 |
| Q2 | 1.34 (0.97, 1.87) | 0.080 | 1.41 (1.16, 1.72) | 0.001 |  |
| Q3 | 0.99 (0.62, 1.58) | 0.982 | 1.66 (1.37, 2.00) | <0.001 |  |
| Q4 | 2.40 (0.71, 8.12) | 0.160 | 1.76 (1.46, 2.12) | <0.001 |  |
| METS-IR | | | | | |
| Q1 | Ref |  | Ref |  | 0.544 |
| Q2 | 1.55 (1.12, 2.14) | 0.009 | 1.30 (1.06, 1.59) | 0.012 |  |
| Q3 | 1.37 (0.83, 2.27) | 0.214 | 1.62 (1.34, 1.96) | <0.001 |  |
| Q4 | 1.57 (0.49, 5.08) | 0.452 | 1.84 (1.52, 2.22) | <0.001 |  |
| AIP | | | | | |
| Q1 | Ref |  | Ref |  | 0.022 |
| Q2 | 1.68 (1.21, 2.35) | 0.002 | 1.37 (1.13, 1.66) | 0.002 |  |
| Q3 | 0.90 (0.56, 1.46) | 0.670 | 1.58 (1.32, 1.90) | <0.001 |  |
| Q4 | 2.22 (0.66, 7.52) | 0.199 | 1.78 (1.49, 2.13) | <0.001 |  |
| Q1: Quartile 1, Q2: Quartile 2, Q3: Quartile 3, Q4: Quartile 4.  Adjusted for age, sex, residence, smoking status, drinking status, marital status, education level, hypertension, hyperuricemia, and CKD.  In the stage 0–1 stratum (n = 171 events), no participant fell into the highest TyG quartile (Q4, n = 0), and estimates for the highest quartiles of the other indices were often imprecise and non-significant, reflecting limited statistical power. The interaction P value was 0.022 for AIP; however, given the imprecise estimates in this stratum, this interaction was not interpreted. | | | | | |

| **Table S11** Association between various IR surrogates and CVD incidence in individuals with CKM syndrome stages 0-3 by Hypertension | | | | | |
| --- | --- | --- | --- | --- | --- |
| **IR surrogates**  **(Quartile)** | **No** | | **Yes** | | ***P* for interaction** |
|  | **HR(95% CI)** | ***P*** | **HR(95% CI)** | ***P*** |  |
| CHG | | | | | |
| Q1 | Ref |  | Ref |  | 0.094 |
| Q2 | 1.29 (1.02, 1.62) | **0.036** | 1.15 (0.89, 1.49) | 0.293 |  |
| Q3 | 1.46 (1.15, 1.85) | **0.002** | 1.79 (1.42, 2.28) | **<0.001** |  |
| Q4 | 1.89 (1.49, 2.40) | **<0.001** | 1.68 (1.33, 2.13) | **<0.001** |  |
| CHG- BMI | | | | | |
| Q1 | Ref |  | Ref |  | 0.337 |
| Q2 | 1.52 (1.22, 1.91) | **<0.001** | 1.12 (0.87, 1.45) | 0.373 |  |
| Q3 | 1.53 (1.21, 1.93) | **<0.001** | 1.29 (1.01, 1.65) | **0.042** |  |
| Q4 | 1.85 (1.45, 2.37) | **<0.001** | 1.65 (1.31, 2.09) | **<0.001** |  |
| CHG- WC | | | | | |
| Q1 | Ref |  | Ref |  | 0.536 |
| Q2 | 1.36 (1.09, 1.70) | **0.006** | 1.36 (1.03, 1.78) | **0.029** |  |
| Q3 | 1.28 (1.01, 1.62) | **0.044** | 1.58 (1.23, 2.04) | **<0.001** |  |
| Q4 | 1.71 (1.34, 2.18) | **<0.001** | 1.80 (1.40, 2.31) | **<0.001** |  |
| CHG- CVAI | | | | | |
| Q1 | Ref |  | Ref |  | 0.531 |
| Q2 | 1.44 (1.14, 1.80) | **0.002** | 1.95 (1.42, 2.67) | **<0.001** |  |
| Q3 | 1.52 (1.19, 1.94) | **0.001** | 1.98 (1.47, 2.67) | **<0.001** |  |
| Q4 | 1.92 (1.48, 2.49) | **<0.001** | 2.41 (1.79, 3.24) | **<0.001** |  |
| CHG- BRI | | | | | |
| Q1 | Ref |  | Ref |  | 0.550 |
| Q2 | 1.20 (0.96, 1.50) | 0.108 | 1.39 (1.05, 1.83) | **0.022** |  |
| Q3 | 1.33 (1.05, 1.67) | **0.016** | 1.75 (1.35, 2.28) | **<0.001** |  |
| Q4 | 1.42 (1.11, 1.81) | **0.006** | 1.73 (1.34, 2.24) | **<0.001** |  |
| CHG- WHtR | | | | | |
| Q1 | Ref |  | Ref |  | 0.433 |
| Q2 | 1.14 (0.91, 1.42) | 0.253 | 1.56 (1.17, 2.08) | **0.002** |  |
| Q3 | 1.40 (1.12, 1.76) | **0.003** | 1.78 (1.36, 2.33) | **<0.001** |  |
| Q4 | 1.46 (1.14, 1.87) | **0.003** | 2.00 (1.54, 2.60) | **<0.001** |  |
| CHG- WWI | | | | | |
| Q1 | Ref |  | Ref |  | 0.297 |
| Q2 | 1.05 (0.83, 1.31) | 0.704 | 1.52 (1.16, 2.00) | 0.002 |  |
| Q3 | 1.24 (0.99, 1.57) | 0.065 | 1.70 (1.31, 2.20) | <0.001 |  |
| Q4 | 1.39 (1.09, 1.77) | 0.009 | 1.85 (1.43, 2.40) | <0.001 |  |
| CHG- ABSI | | | | | |
| Q1 | Ref |  | Ref |  | 0.326 |
| Q2 | 1.16 (0.93, 1.46) | 0.193 | 1.20 (0.93, 1.55) | 0.152 |  |
| Q3 | 1.15 (0.91, 1.46) | 0.235 | 1.53 (1.20, 1.94) | **0.001** |  |
| Q4 | 1.37 (1.07, 1.75) | **0.012** | 1.51 (1.19, 1.91) | **0.001** |  |
| TyG | | | | | |
| Q1 | Ref |  | Ref |  | 0.203 |
| Q2 | 1.32 (1.05, 1.67) | **0.020** | 1.42 (1.11, 1.82) | **0.005** |  |
| Q3 | 1.79 (1.42, 2.26) | **<0.001** | 1.46 (1.15, 1.84) | **0.002** |  |
| Q4 | 1.91 (1.50, 2.44) | **<0.001** | 1.63 (1.30, 2.05) | **<0.001** |  |
| CMI | | | | | |
| Q1 | Ref |  | Ref |  | 0.818 |
| Q2 | 1.28 (1.02, 1.61) | **0.037** | 1.50 (1.16, 1.93) | **0.002** |  |
| Q3 | 1.53 (1.22, 1.93) | **<0.001** | 1.63 (1.28, 2.07) | **<0.001** |  |
| Q4 | 1.70 (1.34, 2.17) | **<0.001** | 1.78 (1.41, 2.26) | **<0.001** |  |
| METS-IR | | | | | |
| Q1 | Ref |  | Ref |  | 0.480 |
| Q2 | 1.44 (1.15, 1.81) | **0.002** | 1.24 (0.95, 1.61) | 0.106 |  |
| Q3 | 1.86 (1.48, 2.34) | **<0.001** | 1.43 (1.12, 1.83) | **0.004** |  |
| Q4 | 1.93 (1.51, 2.48) | **<0.001** | 1.71 (1.35, 2.18) | **<0.001** |  |
| AIP | | | | | |
| Q1 | Ref |  | Ref |  | 0.503 |
| Q2 | 1.35 (1.07, 1.70) | **0.012** | 1.50 (1.18, 1.91) | **0.001** |  |
| Q3 | 1.42 (1.12, 1.80) | **0.004** | 1.59 (1.26, 2.00) | **<0.001** |  |
| Q4 | 1.91 (1.50, 2.42) | **<0.001** | 1.76 (1.40, 2.21) | **<0.001** |  |
| Q1: Quartile 1, Q2: Quartile 2, Q3: Quartile 3, Q4: Quartile 4.  Adjusted for age, sex, residence, smoking status, drinking status, marital status, education level, hyperuricemia, and CKD (hypertension excluded because it is the stratifying variable). | | | | | |

| **Table S12** Association between various IR surrogates and CVD incidence in individuals with CKM syndrome stages 0-3 by Hyperuricemia | | | | | |
| --- | --- | --- | --- | --- | --- |
| **IR surrogates**  **(Quartile)** | **No** | | **Yes** | | ***P* for interaction** |
|  | **HR(95% CI)** | ***P*** | **HR(95% CI)** | ***P*** |  |
| CHG | | | | | |
| Q1 | Ref |  | Ref |  | 0.727 |
| Q2 | 1.24 (1.03, 1.49) | **0.020** | 1.15 (0.65, 2.06) | 0.626 |  |
| Q3 | 1.68 (1.41, 1.99) | **<0.001** | 1.61 (0.94, 2.76) | 0.082 |  |
| Q4 | 1.72 (1.44, 2.05) | **<0.001** | 2.01 (1.20, 3.35) | **0.008** |  |
| CHG- BMI | | | | | |
| Q1 | Ref |  | Ref |  | 0.307 |
| Q2 | 1.36 (1.14, 1.62) | **0.001** | 1.04 (0.57, 1.89) | 0.894 |  |
| Q3 | 1.40 (1.17, 1.67) | **<0.001** | 1.67 (0.98, 2.86) | 0.060 |  |
| Q4 | 1.78 (1.49, 2.12) | **<0.001** | 2.05 (1.22, 3.45) | **0.007** |  |
| CHG- WC | | | | | |
| Q1 | Ref |  | Ref |  | 0.271 |
| Q2 | 1.35 (1.13, 1.62) | **0.001** | 1.52 (0.81, 2.87) | 0.195 |  |
| Q3 | 1.37 (1.15, 1.64) | **0.001** | 2.29 (1.27, 4.12) | **0.006** |  |
| Q4 | 1.71 (1.43, 2.04) | **<0.001** | 2.33 (1.31, 4.13) | **0.004** |  |
| CHG- CVAI | | | | | |
| Q1 | Ref |  | Ref |  | 0.406 |
| Q2 | 1.68 (1.39, 2.02) | **<0.001** | 1.21 (0.60, 2.45) | 0.593 |  |
| Q3 | 1.72 (1.43, 2.08) | **<0.001** | 1.72 (0.91, 3.26) | 0.095 |  |
| Q4 | 2.03 (1.67, 2.48) | **<0.001** | 2.46 (1.33, 4.55) | **0.004** |  |
| CHG- BRI | | | | | |
| Q1 | Ref |  | Ref |  | 0.697 |
| Q2 | 1.23 (1.03, 1.47) | **0.024** | 1.57 (0.87, 2.81) | 0.131 |  |
| Q3 | 1.47 (1.23, 1.76) | **<0.001** | 2.15 (1.24, 3.70) | **0.006** |  |
| Q4 | 1.47 (1.23, 1.77) | **<0.001** | 2.16 (1.27, 3.67) | **0.005** |  |
| CHG- WHtR | | | | | |
| Q1 | Ref |  | Ref |  | 0.413 |
| Q2 | 1.31 (1.09, 1.57) | **0.003** | 1.17 (0.65, 2.14) | 0.598 |  |
| Q3 | 1.50 (1.26, 1.79) | **<0.001** | 2.06 (1.19, 3.55) | **0.010** |  |
| Q4 | 1.66 (1.39, 1.99) | **<0.001** | 1.96 (1.15, 3.35) | **0.014** |  |
| CHG- WWI | | | | | |
| Q1 | Ref |  | Ref |  | 0.982 |
| Q2 | 1.24 (1.03, 1.48) | 0.021 | 1.15 (0.67, 1.97) | 0.612 |  |
| Q3 | 1.43 (1.20, 1.71) | <0.001 | 1.36 (0.82, 2.25) | 0.233 |  |
| Q4 | 1.56 (1.30, 1.88) | <0.001 | 1.54 (0.94, 2.51) | 0.087 |  |
| CHG- ABSI | | | | | |
| Q1 | Ref |  | Ref |  | 0.713 |
| Q2 | 1.20 (1.00, 1.43) | **0.046** | 1.05 (0.62, 1.79) | 0.851 |  |
| Q3 | 1.32 (1.10, 1.57) | **0.002** | 1.45 (0.89, 2.35) | 0.136 |  |
| Q4 | 1.42 (1.19, 1.70) | **<0.001** | 1.44 (0.89, 2.33) | 0.137 |  |
| TyG | | | | | |
| Q1 | Ref |  | Ref |  | 0.933 |
| Q2 | 1.37 (1.15, 1.63) | **<0.001** | 1.87 (0.94, 3.71) | 0.073 |  |
| Q3 | 1.60 (1.35, 1.90) | **<0.001** | 2.19 (1.16, 4.14) | **0.016** |  |
| Q4 | 1.76 (1.47, 2.09) | **<0.001** | 2.30 (1.24, 4.28) | **0.008** |  |
| CMI | | | | | |
| Q1 | Ref |  | Ref |  | 0.966 |
| Q2 | 1.36 (1.14, 1.63) | **0.001** | 1.74 (0.92, 3.28) | 0.089 |  |
| Q3 | 1.56 (1.32, 1.86) | **<0.001** | 1.99 (1.10, 3.61) | **0.023** |  |
| Q4 | 1.72 (1.44, 2.05) | **<0.001** | 2.10 (1.20, 3.68) | **0.010** |  |
| METS-IR | | | | | |
| Q1 | Ref |  | Ref |  | 0.845 |
| Q2 | 1.38 (1.15, 1.65) | **<0.001** | 1.27 (0.67, 2.39) | 0.462 |  |
| Q3 | 1.68 (1.41, 2.00) | **<0.001** | 1.63 (0.91, 2.94) | 0.102 |  |
| Q4 | 1.85 (1.55, 2.21) | **<0.001** | 2.02 (1.16, 3.54) | **0.014** |  |
| AIP | | | | | |
| Q1 | Ref |  | Ref |  | 0.923 |
| Q2 | 1.42 (1.19, 1.69) | **<0.001** | 1.49 (0.82, 2.70) | 0.188 |  |
| Q3 | 1.54 (1.29, 1.83) | **<0.001** | 1.48 (0.85, 2.60) | 0.169 |  |
| Q4 | 1.82 (1.53, 2.16) | **<0.001** | 1.79 (1.05, 3.04) | **0.031** |  |
| Q1: Quartile 1, Q2: Quartile 2, Q3: Quartile 3, Q4: Quartile 4.  Adjusted for age, sex, residence, smoking status, drinking status, marital status, education level, hypertension, and CKD (hyperuricemia excluded because it is the stratifying variable). | | | | | |

**Table S13** Association between various IR surrogates and CVD incidence in individuals with CKM syndrome stages 0-3: a study with follow-up time ≥5 years

|  | **Total,n** | **Event,n** | **Model 1** | | **Model 2** | | **Model 3** | |
| --- | --- | --- | --- | --- | --- | --- | --- | --- |
|  |  |  | **HR(95%CI)** | ***P*** | **HR(95%CI)** | ***P*** | **HR(95%CI)** | ***P*** |
| **CHG** |  |  |  |  |  |  |  |  |
| per 1 SD | 5459 | 1343 | 1.30 (1.24, 1.37) | <0.001 | 1.21 (1.15, 1.28) | <0.001 | 1.19 (1.13, 1.25) | <0.001 |
| Q1 | 1364 | 220 | Ref |  | Ref |  | Ref |  |
| Q2 | 1365 | 291 | 1.38 (1.16, 1.65) | <0.001 | 1.27 (1.06, 1.51) | 0.009 | 1.24 (1.04, 1.47) | 0.019 |
| Q3 | 1365 | 385 | 1.94 (1.65, 2.30) | <0.001 | 1.68 (1.42, 1.99) | <0.001 | 1.61 (1.36, 1.91) | <0.001 |
| Q4 | 1365 | 447 | 2.35 (2.00, 2.77) | <0.001 | 1.88 (1.59, 2.22) | <0.001 | 1.75 (1.48, 2.06) | <0.001 |
| *P*-trend |  |  | <0.001 |  | <0.001 |  | <0.001 |  |
| **CHG-BMI** |  |  |  |  |  |  |  |  |
| per 1 SD | 5459 | 1343 | 1.29 (1.23, 1.36) | <0.001 | 1.25 (1.19, 1.31) | <0.001 | 1.21 (1.14, 1.27) | <0.001 |
| Q1 | 1364 | 225 | Ref |  | Ref |  | Ref |  |
| Q2 | 1365 | 322 | 1.46 (1.23, 1.73) | <0.001 | 1.39 (1.17, 1.65) | <0.001 | 1.34 (1.12, 1.59) | 0.001 |
| Q3 | 1365 | 351 | 1.67 (1.41, 1.97) | <0.001 | 1.55 (1.31, 1.83) | <0.001 | 1.46 (1.23, 1.73) | <0.001 |
| Q4 | 1365 | 445 | 2.21 (1.88, 2.59) | <0.001 | 1.97 (1.68, 2.32) | <0.001 | 1.79 (1.51, 2.12) | <0.001 |
| *P*-trend |  |  | <0.001 |  | <0.001 |  | <0.001 |  |
| **CHG-WC** |  |  |  |  |  |  |  |  |
| per 1 SD | 5459 | 1343 | 1.36 (1.29, 1.43) | <0.001 | 1.24 (1.18, 1.31) | <0.001 | 1.20 (1.14, 1.27) | <0.001 |
| Q1 | 1364 | 214 | Ref |  | Ref |  | Ref |  |
| Q2 | 1365 | 312 | 1.56 (1.31, 1.85) | <0.001 | 1.44 (1.21, 1.71) | <0.001 | 1.39 (1.17, 1.66) | <0.001 |
| Q3 | 1365 | 369 | 1.90 (1.60, 2.25) | <0.001 | 1.56 (1.32, 1.85) | <0.001 | 1.47 (1.24, 1.75) | <0.001 |
| Q4 | 1365 | 448 | 2.44 (2.07, 2.87) | <0.001 | 1.92 (1.62, 2.26) | <0.001 | 1.75 (1.47, 2.07) | <0.001 |
| *P*-trend |  |  | <0.001 |  | <0.001 |  | <0.001 |  |
| **CHG-WHtR** |  |  |  |  |  |  |  |  |
| per 1 SD | 5459 | 1343 | 1.37 (1.30, 1.44) | <0.001 | 1.23 (1.17, 1.30) | <0.001 | 1.19 (1.12, 1.26) | <0.001 |
| Q1 | 1364 | 215 | Ref |  | Ref |  | Ref |  |
| Q2 | 1365 | 291 | 1.42 (1.19, 1.70) | <0.001 | 1.30 (1.09, 1.56) | 0.003 | 1.27 (1.07, 1.52) | 0.008 |
| Q3 | 1365 | 388 | 1.99 (1.68, 2.35) | <0.001 | 1.67 (1.41, 1.98) | <0.001 | 1.57 (1.32, 1.86) | <0.001 |
| Q4 | 1365 | 449 | 2.41 (2.05, 2.84) | <0.001 | 1.82 (1.54, 2.15) | <0.001 | 1.66 (1.39, 1.97) | <0.001 |
| *P*-trend |  |  | <0.001 |  | <0.001 |  | <0.001 |  |
| **CHG-WWI** |  |  |  |  |  |  |  |  |
| per 1 SD | 5459 | 1343 | 1.33 (1.27, 1.40) | <0.001 | 1.17 (1.11, 1.23) | <0.001 | 1.14 (1.07, 1.20) | <0.001 |
| Q1 | 1364 | 222 | Ref |  | Ref |  | Ref |  |
| Q2 | 1365 | 292 | 1.36 (1.14, 1.62) | 0.001 | 1.22 (1.02, 1.45) | 0.029 | 1.19 (1.00, 1.42) | 0.053 |
| Q3 | 1365 | 378 | 1.86 (1.57, 2.19) | <0.001 | 1.49 (1.26, 1.76) | <0.001 | 1.41 (1.19, 1.67) | <0.001 |
| Q4 | 1365 | 451 | 2.39 (2.03, 2.80) | <0.001 | 1.66 (1.40, 1.97) | <0.001 | 1.54 (1.29, 1.83) | <0.001 |
| *P*-trend |  |  | <0.001 |  | <0.001 |  | <0.001 |  |
| **CHG-BRI** |  |  |  |  |  |  |  |  |
| per 1 SD | 5459 | 1343 | 1.31 (1.24, 1.37) | <0.001 | 1.19 (1.13, 1.25) | <0.001 | 1.15 (1.08, 1.21) | <0.001 |
| Q1 | 1364 | 218 | Ref |  | Ref |  | Ref |  |
| Q2 | 1365 | 302 | 1.45 (1.22, 1.72) | <0.001 | 1.30 (1.09, 1.55) | 0.003 | 1.27 (1.06, 1.51) | 0.008 |
| Q3 | 1365 | 384 | 1.97 (1.66, 2.32) | <0.001 | 1.64 (1.39, 1.94) | <0.001 | 1.54 (1.30, 1.83) | <0.001 |
| Q4 | 1365 | 439 | 2.28 (1.94, 2.68) | <0.001 | 1.71 (1.45, 2.03) | <0.001 | 1.55 (1.30, 1.84) | <0.001 |
| *P*-trend |  |  | <0.001 |  | <0.001 |  | <0.001 |  |
| **CHG-ABSI** |  |  |  |  |  |  |  |  |
| per 1 SD | 5459 | 1343 | 1.29 (1.23, 1.35) | <0.001 | 1.14 (1.08, 1.20) | <0.001 | 1.11 (1.05, 1.18) | <0.001 |
| Q1 | 1364 | 229 | Ref |  | Ref |  | Ref |  |
| Q2 | 1365 | 309 | 1.41 (1.19, 1.68) | <0.001 | 1.23 (1.04, 1.46) | 0.018 | 1.20 (1.01, 1.43) | 0.036 |
| Q3 | 1365 | 375 | 1.78 (1.51, 2.10) | <0.001 | 1.43 (1.21, 1.70) | <0.001 | 1.37 (1.16, 1.62) | <0.001 |
| Q4 | 1365 | 430 | 2.20 (1.87, 2.58) | <0.001 | 1.51 (1.28, 1.79) | <0.001 | 1.41 (1.19, 1.68) | <0.001 |
| *P*-trend |  |  | <0.001 |  | <0.001 |  | <0.001 |  |
| **CHG-CVAI** |  |  |  |  |  |  |  |  |
| per 1 SD | 5459 | 1343 | 1.48 (1.41, 1.56) | <0.001 | 1.27 (1.20, 1.35) | <0.001 | 1.23 (1.16, 1.31) | <0.001 |
| Q1 | 1364 | 182 | Ref |  | Ref |  | Ref |  |
| Q2 | 1365 | 305 | 1.84 (1.53, 2.21) | <0.001 | 1.61 (1.34, 1.94) | <0.001 | 1.59 (1.32, 1.91) | <0.001 |
| Q3 | 1365 | 373 | 2.37 (1.98, 2.83) | <0.001 | 1.79 (1.49, 2.15) | <0.001 | 1.68 (1.40, 2.03) | <0.001 |
| Q4 | 1365 | 483 | 3.35 (2.83, 3.98) | <0.001 | 2.24 (1.87, 2.69) | <0.001 | 2.04 (1.69, 2.46) | <0.001 |
| *P*-trend |  |  | <0.001 |  | <0.001 |  | <0.001 |  |
| **TyG** |  |  |  |  |  |  |  |  |
| per 1 SD | 5459 | 1343 | 1.29 (1.23, 1.36) | <0.001 | 1.23 (1.17, 1.29) | <0.001 | 1.19 (1.13, 1.26) | <0.001 |
| Q1 | 1362 | 225 | Ref |  | Ref |  | Ref |  |
| Q2 | 1364 | 306 | 1.46 (1.23, 1.74) | <0.001 | 1.37 (1.15, 1.63) | <0.001 | 1.35 (1.14, 1.61) | 0.001 |
| Q3 | 1368 | 386 | 1.96 (1.67, 2.32) | <0.001 | 1.67 (1.42, 1.97) | <0.001 | 1.59 (1.34, 1.88) | <0.001 |
| Q4 | 1365 | 426 | 2.27 (1.93, 2.66) | <0.001 | 1.86 (1.58, 2.20) | <0.001 | 1.73 (1.46, 2.05) | <0.001 |
| *P*-trend |  |  | <0.001 |  | <0.001 |  | <0.001 |  |
| **AIP** |  |  |  |  |  |  |  |  |
| per 1 SD | 5459 | 1343 | 1.26 (1.20, 1.32) | <0.001 | 1.21 (1.15, 1.28) | <0.001 | 1.18 (1.12, 1.25) | <0.001 |
| Q1 | 1364 | 233 | Ref |  | Ref |  | Ref |  |
| Q2 | 1365 | 314 | 1.46 (1.23, 1.73) | <0.001 | 1.40 (1.18, 1.66) | <0.001 | 1.38 (1.16, 1.63) | <0.001 |
| Q3 | 1365 | 367 | 1.77 (1.50, 2.09) | <0.001 | 1.54 (1.31, 1.82) | <0.001 | 1.48 (1.25, 1.75) | <0.001 |
| Q4 | 1365 | 429 | 2.15 (1.83, 2.52) | <0.001 | 1.85 (1.57, 2.17) | <0.001 | 1.73 (1.47, 2.04) | <0.001 |
| *P*-trend |  |  | <0.001 |  | <0.001 |  | <0.001 |  |
| **METS-IR** |  |  |  |  |  |  |  |  |
| per 1 SD | 5459 | 1343 | 1.29 (1.23, 1.36) | <0.001 | 1.25 (1.19, 1.31) | <0.001 | 1.21 (1.15, 1.28) | <0.001 |
| Q1 | 1364 | 217 | Ref |  | Ref |  | Ref |  |
| Q2 | 1365 | 302 | 1.45 (1.22, 1.73) | <0.001 | 1.39 (1.17, 1.66) | <0.001 | 1.35 (1.13, 1.61) | 0.001 |
| Q3 | 1365 | 389 | 2.01 (1.70, 2.37) | <0.001 | 1.76 (1.49, 2.08) | <0.001 | 1.65 (1.39, 1.96) | <0.001 |
| Q4 | 1365 | 435 | 2.34 (1.99, 2.75) | <0.001 | 2.02 (1.71, 2.38) | <0.001 | 1.85 (1.56, 2.20) | <0.001 |
| *P*-trend |  |  | <0.001 |  | <0.001 |  | <0.001 |  |
| **CMI** |  |  |  |  |  |  |  |  |
| per 1 SD | 5459 | 1343 | 1.08 (1.05, 1.12) | <0.001 | 1.08 (1.04, 1.12) | <0.001 | 1.07 (1.02, 1.11) | 0.002 |
| Q1 | 1364 | 226 | Ref |  | Ref |  | Ref |  |
| Q2 | 1365 | 310 | 1.49 (1.26, 1.77) | <0.001 | 1.41 (1.19, 1.68) | <0.001 | 1.38 (1.16, 1.64) | <0.001 |
| Q3 | 1365 | 384 | 1.94 (1.64, 2.28) | <0.001 | 1.65 (1.39, 1.94) | <0.001 | 1.56 (1.32, 1.85) | <0.001 |
| Q4 | 1365 | 423 | 2.19 (1.86, 2.57) | <0.001 | 1.83 (1.55, 2.16) | <0.001 | 1.70 (1.44, 2.01) | <0.001 |
| *P*-trend |  |  | <0.001 |  | <0.001 |  | <0.001 |  |
| HR Hazard Ratio, CI Confidence Interval  Q1: Quartile 1, Q2: Quartile 2, Q3: Quartile 3, Q4: Quartile 4.  Total n=5459 (participants with follow-up ≥5 years), 1343 events.  Model 1: unadjusted for covariates; Model 2: adjusted for age, sex, residence, smoking status, drinking status, marital status and education level; Model 3: further adjusted for hypertension, hyperuricemia and CKD. | | | | | | | | |

**Table S14** Association between various IR surrogates and CVD incidence in individuals with CKM syndrome stages 0-3: excluding participants taking lipid-lowering and hypoglycemic medications

|  | **Total,n** | **Event,n** | **Model 1** | | **Model 2** | | **Model 3** | |
| --- | --- | --- | --- | --- | --- | --- | --- | --- |
|  |  |  | **HR(95%CI)** | ***P*** | **HR(95%CI)** | ***P*** | **HR(95%CI)** | ***P*** |
| **CHG** |  |  |  |  |  |  |  |  |
| per 1 SD | 5321 | 1287 | 1.29 (1.23, 1.36) | <0.001 | 1.20 (1.14, 1.26) | <0.001 | 1.17 (1.11, 1.24) | <0.001 |
| Q1 | 1330 | 220 | Ref |  | Ref |  | Ref |  |
| Q2 | 1330 | 278 | 1.31 (1.10, 1.57) | 0.003 | 1.18 (0.99, 1.41) | 0.068 | 1.15 (0.96, 1.37) | 0.130 |
| Q3 | 1330 | 363 | 1.80 (1.53, 2.13) | <0.001 | 1.57 (1.32, 1.86) | <0.001 | 1.50 (1.27, 1.78) | <0.001 |
| Q4 | 1331 | 426 | 2.21 (1.88, 2.60) | <0.001 | 1.74 (1.48, 2.06) | <0.001 | 1.62 (1.37, 1.92) | <0.001 |
| *P*-trend |  |  | <0.001 |  | <0.001 |  | <0.001 |  |
| **CHG-BMI** |  |  |  |  |  |  |  |  |
| per 1 SD | 5321 | 1287 | 1.29 (1.22, 1.35) | <0.001 | 1.24 (1.18, 1.31) | <0.001 | 1.20 (1.14, 1.27) | <0.001 |
| Q1 | 1330 | 217 | Ref |  | Ref |  | Ref |  |
| Q2 | 1330 | 315 | 1.48 (1.24, 1.76) | <0.001 | 1.40 (1.18, 1.67) | <0.001 | 1.35 (1.14, 1.61) | 0.001 |
| Q3 | 1330 | 322 | 1.56 (1.31, 1.85) | <0.001 | 1.45 (1.22, 1.73) | <0.001 | 1.37 (1.15, 1.63) | <0.001 |
| Q4 | 1331 | 433 | 2.20 (1.87, 2.59) | <0.001 | 1.98 (1.68, 2.33) | <0.001 | 1.81 (1.53, 2.15) | <0.001 |
| *P*-trend |  |  | <0.001 |  | <0.001 |  | <0.001 |  |
| **CHG-WC** |  |  |  |  |  |  |  |  |
| per 1 SD | 5321 | 1287 | 1.34 (1.27, 1.41) | <0.001 | 1.23 (1.16, 1.30) | <0.001 | 1.19 (1.12, 1.26) | <0.001 |
| Q1 | 1330 | 206 | Ref |  | Ref |  | Ref |  |
| Q2 | 1330 | 303 | 1.57 (1.31, 1.87) | <0.001 | 1.45 (1.21, 1.73) | <0.001 | 1.40 (1.17, 1.68) | <0.001 |
| Q3 | 1330 | 349 | 1.85 (1.56, 2.20) | <0.001 | 1.51 (1.27, 1.80) | <0.001 | 1.43 (1.20, 1.70) | <0.001 |
| Q4 | 1331 | 429 | 2.41 (2.04, 2.85) | <0.001 | 1.90 (1.61, 2.25) | <0.001 | 1.74 (1.46, 2.07) | <0.001 |
| *P*-trend |  |  | <0.001 |  | <0.001 |  | <0.001 |  |
| **CHG-WHtR** |  |  |  |  |  |  |  |  |
| per 1 SD | 5321 | 1287 | 1.35 (1.28, 1.43) | <0.001 | 1.22 (1.15, 1.29) | <0.001 | 1.18 (1.11, 1.25) | <0.001 |
| Q1 | 1330 | 207 | Ref |  | Ref |  | Ref |  |
| Q2 | 1330 | 291 | 1.49 (1.24, 1.78) | <0.001 | 1.35 (1.13, 1.61) | 0.001 | 1.32 (1.10, 1.58) | 0.002 |
| Q3 | 1330 | 365 | 1.93 (1.62, 2.28) | <0.001 | 1.62 (1.36, 1.92) | <0.001 | 1.52 (1.28, 1.81) | <0.001 |
| Q4 | 1331 | 424 | 2.35 (1.99, 2.78) | <0.001 | 1.75 (1.47, 2.08) | <0.001 | 1.59 (1.33, 1.90) | <0.001 |
| *P*-trend |  |  | <0.001 |  | <0.001 |  | <0.001 |  |
| **CHG-WWI** |  |  |  |  |  |  |  |  |
| per 1 SD | 5321 | 1287 | 1.32 (1.25, 1.39) | <0.001 | 1.15 (1.08, 1.22) | <0.001 | 1.12 (1.05, 1.18) | <0.001 |
| Q1 | 1330 | 217 | Ref |  | Ref |  | Ref |  |
| Q2 | 1330 | 286 | 1.37 (1.15, 1.64) | <0.001 | 1.23 (1.03, 1.47) | 0.022 | 1.20 (1.01, 1.44) | 0.041 |
| Q3 | 1330 | 354 | 1.77 (1.50, 2.10) | <0.001 | 1.42 (1.20, 1.69) | <0.001 | 1.34 (1.13, 1.60) | 0.001 |
| Q4 | 1331 | 430 | 2.31 (1.97, 2.72) | <0.001 | 1.60 (1.35, 1.90) | <0.001 | 1.48 (1.24, 1.77) | <0.001 |
| *P*-trend |  |  | <0.001 |  | <0.001 |  | <0.001 |  |
| **CHG-BRI** |  |  |  |  |  |  |  |  |
| per 1 SD | 5321 | 1287 | 1.30 (1.24, 1.37) | <0.001 | 1.18 (1.12, 1.24) | <0.001 | 1.14 (1.08, 1.21) | <0.001 |
| Q1 | 1330 | 207 | Ref |  | Ref |  | Ref |  |
| Q2 | 1330 | 296 | 1.51 (1.26, 1.80) | <0.001 | 1.34 (1.12, 1.60) | 0.001 | 1.31 (1.09, 1.56) | 0.003 |
| Q3 | 1330 | 362 | 1.94 (1.63, 2.30) | <0.001 | 1.62 (1.36, 1.93) | <0.001 | 1.52 (1.28, 1.81) | <0.001 |
| Q4 | 1331 | 422 | 2.32 (1.96, 2.74) | <0.001 | 1.73 (1.46, 2.06) | <0.001 | 1.57 (1.32, 1.88) | <0.001 |
| *P*-trend |  |  | <0.001 |  | <0.001 |  | <0.001 |  |
| **CHG-ABSI** |  |  |  |  |  |  |  |  |
| per 1 SD | 5321 | 1287 | 1.27 (1.21, 1.34) | <0.001 | 1.11 (1.05, 1.18) | <0.001 | 1.09 (1.03, 1.15) | 0.002 |
| Q1 | 1330 | 227 | Ref |  | Ref |  | Ref |  |
| Q2 | 1330 | 299 | 1.40 (1.18, 1.67) | <0.001 | 1.22 (1.02, 1.45) | 0.026 | 1.19 (1.00, 1.42) | 0.049 |
| Q3 | 1330 | 356 | 1.72 (1.46, 2.03) | <0.001 | 1.36 (1.15, 1.62) | <0.001 | 1.30 (1.10, 1.55) | 0.002 |
| Q4 | 1331 | 405 | 2.09 (1.78, 2.46) | <0.001 | 1.44 (1.21, 1.71) | <0.001 | 1.35 (1.14, 1.61) | 0.001 |
| *P*-trend |  |  | <0.001 |  | <0.001 |  | <0.001 |  |
| **CHG-CVAI** |  |  |  |  |  |  |  |  |
| per 1 SD | 5321 | 1287 | 1.47 (1.40, 1.55) | <0.001 | 1.26 (1.19, 1.34) | <0.001 | 1.22 (1.15, 1.30) | <0.001 |
| Q1 | 1330 | 174 | Ref |  | Ref |  | Ref |  |
| Q2 | 1330 | 293 | 1.85 (1.53, 2.23) | <0.001 | 1.61 (1.34, 1.95) | <0.001 | 1.59 (1.31, 1.92) | <0.001 |
| Q3 | 1330 | 356 | 2.35 (1.96, 2.82) | <0.001 | 1.77 (1.47, 2.14) | <0.001 | 1.67 (1.39, 2.02) | <0.001 |
| Q4 | 1331 | 464 | 3.39 (2.84, 4.03) | <0.001 | 2.25 (1.87, 2.71) | <0.001 | 2.06 (1.70, 2.50) | <0.001 |
| *P*-trend |  |  | <0.001 |  | <0.001 |  | <0.001 |  |
| **TyG** |  |  |  |  |  |  |  |  |
| per 1 SD | 5321 | 1287 | 1.27 (1.21, 1.34) | <0.001 | 1.21 (1.15, 1.28) | <0.001 | 1.18 (1.12, 1.25) | <0.001 |
| Q1 | 1329 | 222 | Ref |  | Ref |  | Ref |  |
| Q2 | 1330 | 298 | 1.45 (1.22, 1.72) | <0.001 | 1.36 (1.14, 1.61) | 0.001 | 1.34 (1.12, 1.59) | 0.001 |
| Q3 | 1330 | 364 | 1.86 (1.57, 2.20) | <0.001 | 1.59 (1.34, 1.88) | <0.001 | 1.52 (1.28, 1.80) | <0.001 |
| Q4 | 1332 | 403 | 2.17 (1.84, 2.56) | <0.001 | 1.79 (1.52, 2.11) | <0.001 | 1.67 (1.41, 1.98) | <0.001 |
| *P*-trend |  |  | <0.001 |  | <0.001 |  | <0.001 |  |
| **AIP** |  |  |  |  |  |  |  |  |
| per 1 SD | 5321 | 1287 | 1.25 (1.19, 1.31) | <0.001 | 1.20 (1.14, 1.27) | <0.001 | 1.18 (1.11, 1.24) | <0.001 |
| Q1 | 1330 | 224 | Ref |  | Ref |  | Ref |  |
| Q2 | 1330 | 306 | 1.49 (1.26, 1.77) | <0.001 | 1.43 (1.20, 1.70) | <0.001 | 1.41 (1.18, 1.67) | <0.001 |
| Q3 | 1330 | 356 | 1.78 (1.51, 2.11) | <0.001 | 1.57 (1.33, 1.86) | <0.001 | 1.51 (1.27, 1.78) | <0.001 |
| Q4 | 1331 | 401 | 2.09 (1.78, 2.47) | <0.001 | 1.80 (1.53, 2.13) | <0.001 | 1.69 (1.43, 2.01) | <0.001 |
| *P*-trend |  |  | <0.001 |  | <0.001 |  | <0.001 |  |
| **METS-IR** |  |  |  |  |  |  |  |  |
| per 1 SD | 5321 | 1287 | 1.27 (1.21, 1.33) | <0.001 | 1.23 (1.17, 1.29) | <0.001 | 1.19 (1.13, 1.26) | <0.001 |
| Q1 | 1330 | 212 | Ref |  | Ref |  | Ref |  |
| Q2 | 1330 | 289 | 1.40 (1.17, 1.67) | <0.001 | 1.34 (1.13, 1.61) | 0.001 | 1.30 (1.09, 1.56) | 0.004 |
| Q3 | 1330 | 379 | 1.99 (1.68, 2.35) | <0.001 | 1.73 (1.46, 2.06) | <0.001 | 1.63 (1.37, 1.93) | <0.001 |
| Q4 | 1331 | 407 | 2.19 (1.86, 2.59) | <0.001 | 1.91 (1.61, 2.26) | <0.001 | 1.75 (1.47, 2.09) | <0.001 |
| P-trend |  |  | <0.001 |  | <0.001 |  | <0.001 |  |
| **CMI** |  |  |  |  |  |  |  |  |
| per 1 SD | 5321 | 1287 | 1.07 (1.04, 1.11) | <0.001 | 1.07 (1.03, 1.11) | 0.001 | 1.06 (1.02, 1.10) | 0.008 |
| Q1 | 1330 | 215 | Ref |  | Ref |  | Ref |  |
| Q2 | 1330 | 304 | 1.54 (1.29, 1.83) | <0.001 | 1.45 (1.22, 1.73) | <0.001 | 1.42 (1.20, 1.70) | <0.001 |
| Q3 | 1330 | 365 | 1.92 (1.62, 2.27) | <0.001 | 1.63 (1.37, 1.93) | <0.001 | 1.55 (1.30, 1.84) | <0.001 |
| Q4 | 1331 | 403 | 2.20 (1.86, 2.59) | <0.001 | 1.84 (1.56, 2.18) | <0.001 | 1.71 (1.44, 2.04) | <0.001 |
| *P*-trend |  |  | <0.001 |  | <0.001 |  | <0.001 |  |
| HR Hazard Ratio, CI Confidence Interval  Q1: Quartile 1, Q2: Quartile 2, Q3: Quartile 3, Q4: Quartile 4.  Total n=5321 (excluding 304 participants taking lipid-lowering or hypoglycemic medications), 1287 events.  Model 1: unadjusted for covariates; Model 2: adjusted for age, sex, residence, smoking status, drinking status, marital status and education level; Model 3: further adjusted for hypertension, hyperuricemia and CKD. | | | | | | | | |

**Table S15** Association between tertiles of various IR surrogates and CVD incidence in individuals with CKM syndrome stages 0-3

|  | **Total,n** | **Event,n** | **Model 1** | | **Model 2** | | **Model 3** | |
| --- | --- | --- | --- | --- | --- | --- | --- | --- |
|  |  |  | **HR(95%CI)** | ***P*** | **HR(95%CI)** | ***P*** | **HR(95%CI)** | ***P*** |
| **CHG** |  |  |  |  |  |  |  |  |
| per 1 SD | 5625 | 1407 | 1.32 (1.26, 1.38) | <0.001 | 1.23 (1.17, 1.29) | <0.001 | 1.20 (1.14, 1.26) | <0.001 |
| T1 | 1875 | 321 | Ref |  | Ref |  | Ref |  |
| T2 | 1875 | 462 | 1.53 (1.33, 1.76) | <0.001 | 1.36 (1.18, 1.57) | <0.001 | 1.33 (1.15, 1.53) | <0.001 |
| T3 | 1875 | 624 | 2.27 (1.98, 2.60) | <0.001 | 1.84 (1.60, 2.11) | <0.001 | 1.72 (1.50, 1.98) | <0.001 |
| *P*-trend |  |  | <0.001 |  | <0.001 |  | <0.001 |  |
| **CHG-BMI** |  |  |  |  |  |  |  |  |
| per 1 SD | 5625 | 1407 | 1.30 (1.24, 1.37) | <0.001 | 1.25 (1.19, 1.32) | <0.001 | 1.21 (1.15, 1.28) | <0.001 |
| T1 | 1875 | 339 | Ref |  | Ref |  | Ref |  |
| T2 | 1875 | 468 | 1.44 (1.25, 1.66) | <0.001 | 1.34 (1.16, 1.54) | <0.001 | 1.28 (1.11, 1.48) | 0.001 |
| T3 | 1875 | 600 | 1.98 (1.73, 2.26) | <0.001 | 1.77 (1.54, 2.02) | <0.001 | 1.62 (1.41, 1.87) | <0.001 |
| *P*-trend |  |  | <0.001 |  | <0.001 |  | <0.001 |  |
| **CHG-WC** |  |  |  |  |  |  |  |  |
| per 1 SD | 5625 | 1407 | 1.37 (1.30, 1.44) | <0.001 | 1.25 (1.19, 1.32) | <0.001 | 1.21 (1.14, 1.28) | <0.001 |
| T1 | 1875 | 314 | Ref |  | Ref |  | Ref |  |
| T2 | 1875 | 479 | 1.64 (1.42, 1.89) | <0.001 | 1.42 (1.23, 1.64) | <0.001 | 1.37 (1.19, 1.58) | <0.001 |
| T3 | 1875 | 614 | 2.27 (1.98, 2.60) | <0.001 | 1.81 (1.57, 2.08) | <0.001 | 1.67 (1.44, 1.92) | <0.001 |
| *P*-trend |  |  | <0.001 |  | <0.001 |  | <0.001 |  |
| **CHG-WHtR** |  |  |  |  |  |  |  |  |
| per 1 SD | 5625 | 1407 | 1.38 (1.31, 1.45) | <0.001 | 1.24 (1.17, 1.31) | <0.001 | 1.20 (1.13, 1.26) | <0.001 |
| T1 | 1875 | 317 | Ref |  | Ref |  | Ref |  |
| T2 | 1875 | 468 | 1.58 (1.37, 1.83) | <0.001 | 1.39 (1.20, 1.60) | <0.001 | 1.34 (1.16, 1.54) | <0.001 |
| T3 | 1875 | 622 | 2.28 (1.99, 2.61) | <0.001 | 1.75 (1.52, 2.02) | <0.001 | 1.61 (1.39, 1.86) | <0.001 |
| *P*-trend |  |  | <0.001 |  | <0.001 |  | <0.001 |  |
| **CHG-WWI** |  |  |  |  |  |  |  |  |
| per 1 SD | 5625 | 1407 | 1.35 (1.28, 1.41) | <0.001 | 1.18 (1.12, 1.24) | <0.001 | 1.15 (1.08, 1.21) | <0.001 |
| T1 | 1875 | 328 | Ref |  | Ref |  | Ref |  |
| T2 | 1875 | 483 | 1.55 (1.35, 1.78) | <0.001 | 1.36 (1.18, 1.56) | <0.001 | 1.31 (1.14, 1.51) | <0.001 |
| T3 | 1875 | 596 | 2.12 (1.85, 2.43) | <0.001 | 1.54 (1.33, 1.77) | <0.001 | 1.43 (1.24, 1.65) | <0.001 |
| *P*-trend |  |  | <0.001 |  | <0.001 |  | <0.001 |  |
| **CHG-BRI** |  |  |  |  |  |  |  |  |
| per 1 SD | 5625 | 1407 | 1.32 (1.26, 1.38) | <0.001 | 1.19 (1.13, 1.26) | <0.001 | 1.15 (1.09, 1.22) | <0.001 |
| T1 | 1875 | 327 | Ref |  | Ref |  | Ref |  |
| T2 | 1875 | 475 | 1.55 (1.34, 1.78) | <0.001 | 1.38 (1.20, 1.59) | <0.001 | 1.33 (1.15, 1.53) | <0.001 |
| T3 | 1875 | 605 | 2.11 (1.84, 2.41) | <0.001 | 1.63 (1.42, 1.88) | <0.001 | 1.49 (1.29, 1.72) | <0.001 |
| *P*-trend |  |  | <0.001 |  | <0.001 |  | <0.001 |  |
| **CHG-ABSI** |  |  |  |  |  |  |  |  |
| per 1 SD | 5625 | 1407 | 1.30 (1.24, 1.37) | <0.001 | 1.15 (1.09, 1.21) | <0.001 | 1.12 (1.06, 1.18) | <0.001 |
| T1 | 1875 | 336 | Ref |  | Ref |  | Ref |  |
| T2 | 1875 | 485 | 1.54 (1.34, 1.77) | <0.001 | 1.33 (1.15, 1.53) | <0.001 | 1.30 (1.13, 1.49) | <0.001 |
| T3 | 1875 | 586 | 2.04 (1.78, 2.33) | <0.001 | 1.49 (1.29, 1.72) | <0.001 | 1.41 (1.22, 1.62) | <0.001 |
| *P*-trend |  |  | <0.001 |  | <0.001 |  | <0.001 |  |
| **CHG-CVAI** |  |  |  |  |  |  |  |  |
| per 1 SD | 5625 | 1407 | 1.50 (1.42, 1.57) | <0.001 | 1.28 (1.21, 1.36) | <0.001 | 1.24 (1.17, 1.31) | <0.001 |
| T1 | 1875 | 286 | Ref |  | Ref |  | Ref |  |
| T2 | 1875 | 467 | 1.82 (1.57, 2.11) | <0.001 | 1.50 (1.29, 1.75) | <0.001 | 1.45 (1.25, 1.69) | <0.001 |
| T3 | 1875 | 654 | 2.86 (2.49, 3.29) | <0.001 | 1.97 (1.69, 2.28) | <0.001 | 1.80 (1.55, 2.11) | <0.001 |
| *P*-trend |  |  | <0.001 |  | <0.001 |  | <0.001 |  |
| **TyG** |  |  |  |  |  |  |  |  |
| per 1 SD | 5625 | 1407 | 1.31 (1.25, 1.37) | <0.001 | 1.23 (1.17, 1.30) | <0.001 | 1.20 (1.14, 1.26) | <0.001 |
| T1 | 1875 | 339 | Ref |  | Ref |  | Ref |  |
| T2 | 1876 | 473 | 1.53 (1.33, 1.76) | <0.001 | 1.37 (1.19, 1.58) | <0.001 | 1.33 (1.15, 1.53) | <0.001 |
| T3 | 1874 | 595 | 2.11 (1.85, 2.41) | <0.001 | 1.74 (1.51, 1.99) | <0.001 | 1.62 (1.41, 1.87) | <0.001 |
| *P*-trend |  |  | <0.001 |  | <0.001 |  | <0.001 |  |
| **AIP** |  |  |  |  |  |  |  |  |
| per 1 SD | 5625 | 1407 | 1.27 (1.21, 1.33) | <0.001 | 1.22 (1.16, 1.28) | <0.001 | 1.19 (1.13, 1.25) | <0.001 |
| T1 | 1875 | 337 | Ref |  | Ref |  | Ref |  |
| T2 | 1875 | 490 | 1.60 (1.39, 1.84) | <0.001 | 1.50 (1.30, 1.72) | <0.001 | 1.46 (1.27, 1.68) | <0.001 |
| T3 | 1875 | 580 | 1.98 (1.73, 2.27) | <0.001 | 1.71 (1.49, 1.96) | <0.001 | 1.61 (1.40, 1.85) | <0.001 |
| *P*-trend |  |  | <0.001 |  | <0.001 |  | <0.001 |  |
| **METS-IR** |  |  |  |  |  |  |  |  |
| per 1 SD | 5625 | 1407 | 1.31 (1.25, 1.37) | <0.001 | 1.25 (1.19, 1.31) | <0.001 | 1.22 (1.15, 1.28) | <0.001 |
| T1 | 1875 | 325 | Ref |  | Ref |  | Ref |  |
| T2 | 1875 | 494 | 1.65 (1.43, 1.90) | <0.001 | 1.49 (1.30, 1.72) | <0.001 | 1.42 (1.23, 1.64) | <0.001 |
| T3 | 1875 | 588 | 2.10 (1.83, 2.41) | <0.001 | 1.79 (1.56, 2.06) | <0.001 | 1.65 (1.43, 1.90) | <0.001 |
| *P*-trend |  |  | <0.001 |  | <0.001 |  | <0.001 |  |
| **CMI** |  |  |  |  |  |  |  |  |
| per 1 SD | 5625 | 1407 | 1.08 (1.05, 1.12) | <0.001 | 1.08 (1.04, 1.12) | <0.001 | 1.07 (1.03, 1.11) | 0.001 |
| T1 | 1875 | 331 | Ref |  | Ref |  | Ref |  |
| T2 | 1875 | 488 | 1.62 (1.41, 1.87) | <0.001 | 1.49 (1.29, 1.71) | <0.001 | 1.45 (1.26, 1.66) | <0.001 |
| T3 | 1875 | 588 | 2.05 (1.80, 2.35) | <0.001 | 1.72 (1.50, 1.97) | <0.001 | 1.61 (1.40, 1.85) | <0.001 |
| *P*-trend |  |  | <0.001 |  | <0.001 |  | <0.001 |  |
| HR Hazard Ratio, CI Confidence Interval  T1: Tertile 1, T2: Tertile 2, T3: Tertile 3.  Total n=5625 (tertile analysis), 1407 events.  Model 1: unadjusted for covariates; Model 2: adjusted for age, sex, residence, smoking status, drinking status, marital status and education level; Model 3: further adjusted for hypertension, hyperuricemia and CKD. | | | | | | | | |

**Table S16** Competing-risk analysis of the associations between 12 IR surrogates and CVD, treating non-cardiovascular death as a competing event

|  | **Model 1** | | **Model 2** | | **Model 3** | |
| --- | --- | --- | --- | --- | --- | --- |
|  | **SHR (95%CI)** | ***P*** | **SHR**  **(95%CI)** | ***P*** | **SHR (95%CI)** | ***P*** |
| **CHG** |  |  |  |  |  |  |
| per 1 SD | 1.30 (1.24, 1.36) | <0.001 | 1.22 (1.16, 1.28) | <0.001 | 1.19 (1.13, 1.25) | <0.001 |
| Q1 | Ref |  | Ref |  | Ref |  |
| Q2 | 1.39 (1.17, 1.65) | <0.001 | 1.28 (1.08, 1.52) | 0.005 | 1.26 (1.06, 1.49) | 0.010 |
| Q3 | 2.05 (1.75, 2.41) | <0.001 | 1.81 (1.54, 2.13) | <0.001 | 1.74 (1.48, 2.05) | <0.001 |
| Q4 | 2.36 (2.02, 2.77) | <0.001 | 1.95 (1.66, 2.29) | <0.001 | 1.82 (1.55, 2.15) | <0.001 |
| *P*-trend |  | <0.001 |  | <0.001 |  | <0.001 |
| **CHG-BMI** |  |  |  |  |  |  |
| per 1 SD | 1.30 (1.24, 1.36) | <0.001 | 1.25 (1.19, 1.32) | <0.001 | 1.22 (1.16, 1.28) | <0.001 |
| Q1 | Ref |  | Ref |  | Ref |  |
| Q2 | 1.48 (1.25, 1.74) | <0.001 | 1.41 (1.19, 1.67) | <0.001 | 1.36 (1.15, 1.61) | <0.001 |
| Q3 | 1.65 (1.40, 1.95) | <0.001 | 1.51 (1.28, 1.79) | <0.001 | 1.43 (1.21, 1.70) | <0.001 |
| Q4 | 2.29 (1.96, 2.68) | <0.001 | 2.05 (1.75, 2.40) | <0.001 | 1.88 (1.59, 2.21) | <0.001 |
| *P*-trend |  | <0.001 |  | <0.001 |  | <0.001 |
| **CHG-WC** |  |  |  |  |  |  |
| per 1 SD | 1.36 (1.30, 1.43) | <0.001 | 1.26 (1.19, 1.33) | <0.001 | 1.22 (1.16, 1.29) | <0.001 |
| Q1 | Ref |  | Ref |  | Ref |  |
| Q2 | 1.51 (1.28, 1.79) | <0.001 | 1.40 (1.18, 1.65) | <0.001 | 1.36 (1.14, 1.61) | <0.001 |
| Q3 | 1.88 (1.59, 2.21) | <0.001 | 1.57 (1.33, 1.85) | <0.001 | 1.48 (1.25, 1.75) | <0.001 |
| Q4 | 2.41 (2.06, 2.82) | <0.001 | 1.94 (1.65, 2.28) | <0.001 | 1.77 (1.50, 2.10) | <0.001 |
| *P*-trend |  | <0.001 |  | <0.001 |  | <0.001 |
| **CHG-WHtR** |  |  |  |  |  |  |
| per 1 SD | 1.36 (1.30, 1.43) | <0.001 | 1.24 (1.18, 1.31) | <0.001 | 1.20 (1.14, 1.27) | <0.001 |
| Q1 | Ref |  | Ref |  | Ref |  |
| Q2 | 1.45 (1.22, 1.72) | <0.001 | 1.33 (1.12, 1.58) | 0.001 | 1.30 (1.09, 1.54) | 0.003 |
| Q3 | 1.97 (1.67, 2.31) | <0.001 | 1.66 (1.41, 1.96) | <0.001 | 1.57 (1.33, 1.86) | <0.001 |
| Q4 | 2.45 (2.09, 2.87) | <0.001 | 1.89 (1.60, 2.23) | <0.001 | 1.73 (1.46, 2.05) | <0.001 |
| *P*-trend |  | <0.001 |  | <0.001 |  | <0.001 |
| **CHG-WWI** |  |  |  |  |  |  |
| per 1 SD | 1.32 (1.26, 1.39) | <0.001 | 1.18 (1.12, 1.24) | <0.001 | 1.15 (1.09, 1.21) | <0.001 |
| Q1 | Ref |  | Ref |  | Ref |  |
| Q2 | 1.41 (1.19, 1.67) | <0.001 | 1.28 (1.08, 1.52) | 0.005 | 1.25 (1.05, 1.48) | 0.011 |
| Q3 | 1.91 (1.62, 2.24) | <0.001 | 1.56 (1.32, 1.84) | <0.001 | 1.48 (1.25, 1.74) | <0.001 |
| Q4 | 2.38 (2.03, 2.78) | <0.001 | 1.74 (1.47, 2.06) | <0.001 | 1.61 (1.36, 1.92) | <0.001 |
| *P*-trend |  | <0.001 |  | <0.001 |  | <0.001 |
| **CHG-BRI** |  |  |  |  |  |  |
| per 1 SD | 1.30 (1.25, 1.37) | <0.001 | 1.19 (1.14, 1.26) | <0.001 | 1.16 (1.10, 1.22) | <0.001 |
| Q1 | Ref |  | Ref |  | Ref |  |
| Q2 | 1.46 (1.23, 1.73) | <0.001 | 1.32 (1.12, 1.57) | 0.001 | 1.28 (1.08, 1.52) | 0.004 |
| Q3 | 1.92 (1.64, 2.26) | <0.001 | 1.62 (1.37, 1.91) | <0.001 | 1.52 (1.29, 1.80) | <0.001 |
| Q4 | 2.28 (1.95, 2.67) | <0.001 | 1.75 (1.48, 2.07) | <0.001 | 1.59 (1.35, 1.89) | <0.001 |
| *P*-trend |  | <0.001 |  | <0.001 |  | <0.001 |
| **CHG-ABSI** |  |  |  |  |  |  |
| per 1 SD | 1.28 (1.22, 1.35) | <0.001 | 1.15 (1.09, 1.21) | <0.001 | 1.13 (1.07, 1.19) | <0.001 |
| Q1 | Ref |  | Ref |  | Ref |  |
| Q2 | 1.38 (1.17, 1.63) | <0.001 | 1.21 (1.02, 1.43) | 0.027 | 1.18 (0.99, 1.39) | 0.059 |
| Q3 | 1.75 (1.49, 2.05) | <0.001 | 1.44 (1.22, 1.70) | <0.001 | 1.38 (1.17, 1.62) | <0.001 |
| Q4 | 2.14 (1.83, 2.51) | <0.001 | 1.55 (1.31, 1.83) | <0.001 | 1.45 (1.22, 1.72) | <0.001 |
| *P*-trend |  | <0.001 |  | <0.001 |  | <0.001 |
| **CHG-CVAI** |  |  |  |  |  |  |
| per 1 SD | 1.48 (1.41, 1.55) | <0.001 | 1.30 (1.23, 1.37) | <0.001 | 1.26 (1.19, 1.33) | <0.001 |
| Q1 | Ref |  | Ref |  | Ref |  |
| Q2 | 1.89 (1.58, 2.26) | <0.001 | 1.66 (1.38, 1.98) | <0.001 | 1.63 (1.36, 1.95) | <0.001 |
| Q3 | 2.42 (2.03, 2.87) | <0.001 | 1.87 (1.57, 2.24) | <0.001 | 1.76 (1.47, 2.11) | <0.001 |
| Q4 | 3.37 (2.85, 3.98) | <0.001 | 2.36 (1.98, 2.82) | <0.001 | 2.17 (1.81, 2.60) | <0.001 |
| *P*-trend |  | <0.001 |  | <0.001 |  | <0.001 |
| **TyG** |  |  |  |  |  |  |
| per 1 SD | 1.29 (1.23, 1.35) | <0.001 | 1.22 (1.16, 1.28) | <0.001 | 1.19 (1.13, 1.25) | <0.001 |
| Q1 | Ref |  | Ref |  | Ref |  |
| Q2 | 1.50 (1.27, 1.77) | <0.001 | 1.41 (1.19, 1.67) | <0.001 | 1.38 (1.17, 1.64) | <0.001 |
| Q3 | 1.99 (1.70, 2.34) | <0.001 | 1.71 (1.46, 2.01) | <0.001 | 1.63 (1.38, 1.92) | <0.001 |
| Q4 | 2.27 (1.94, 2.66) | <0.001 | 1.88 (1.60, 2.21) | <0.001 | 1.75 (1.49, 2.07) | <0.001 |
| *P*-trend |  | <0.001 |  | <0.001 |  | <0.001 |
| **AIP** |  |  |  |  |  |  |
| per 1 SD | 1.27 (1.21, 1.33) | <0.001 | 1.22 (1.16, 1.28) | <0.001 | 1.19 (1.14, 1.25) | <0.001 |
| Q1 | Ref |  | Ref |  | Ref |  |
| Q2 | 1.51 (1.28, 1.78) | <0.001 | 1.43 (1.21, 1.69) | <0.001 | 1.41 (1.19, 1.66) | <0.001 |
| Q3 | 1.83 (1.56, 2.15) | <0.001 | 1.59 (1.36, 1.87) | <0.001 | 1.53 (1.30, 1.80) | <0.001 |
| Q4 | 2.22 (1.90, 2.59) | <0.001 | 1.91 (1.63, 2.24) | <0.001 | 1.80 (1.53, 2.12) | <0.001 |
| *P*-trend |  | <0.001 |  | <0.001 |  | <0.001 |
| **METS-IR** |  |  |  |  |  |  |
| per 1 SD | 1.30 (1.24, 1.36) | <0.001 | 1.24 (1.19, 1.30) | <0.001 | 1.21 (1.15, 1.27) | <0.001 |
| Q1 | Ref |  | Ref |  | Ref |  |
| Q2 | 1.48 (1.25, 1.75) | <0.001 | 1.42 (1.20, 1.69) | <0.001 | 1.38 (1.16, 1.64) | <0.001 |
| Q3 | 2.02 (1.72, 2.38) | <0.001 | 1.77 (1.50, 2.09) | <0.001 | 1.67 (1.41, 1.97) | <0.001 |
| Q4 | 2.36 (2.01, 2.76) | <0.001 | 2.04 (1.74, 2.40) | <0.001 | 1.88 (1.59, 2.23) | <0.001 |
| *P*-trend |  | <0.001 |  | <0.001 |  | <0.001 |
| **CMI** |  |  |  |  |  |  |
| per 1 SD | 1.08 (1.04, 1.13) | <0.001 | 1.08 (1.04, 1.12) | <0.001 | 1.07 (1.03, 1.11) | <0.001 |
| Q1 | Ref |  | Ref |  | Ref |  |
| Q2 | 1.49 (1.26, 1.77) | <0.001 | 1.40 (1.19, 1.66) | <0.001 | 1.37 (1.16, 1.62) | <0.001 |
| Q3 | 1.96 (1.67, 2.30) | <0.001 | 1.67 (1.42, 1.96) | <0.001 | 1.59 (1.35, 1.87) | <0.001 |
| Q4 | 2.22 (1.89, 2.60) | <0.001 | 1.86 (1.58, 2.18) | <0.001 | 1.73 (1.47, 2.04) | <0.001 |
| *P*-trend |  | <0.001 |  | <0.001 |  | <0.001 |
| SHR, subdistribution hazard ratio; CI, confidence interval.  Fine–Gray competing-risk regression with non-cardiovascular death as the competing event (n = 247).  Models 1–3 were adjusted as described in Table 3; continuous variables are per 1-SD increment. | | | | | | |

| **Table S17** Fine–Gray competing-risk analysis of the associations between IR surrogates and CVD by age, treating non-cardiovascular death as a competing event | | | | | |
| --- | --- | --- | --- | --- | --- |
| **IR surrogates**  **(Quartile)** | **Age<60** | | **Age>=60** | | ***P* for interaction** |
|  | **SHR (95% CI)** | ***P*** | **SHR (95% CI)** | ***P*** |  |
| CHG | | | | | |
| Q1 | Ref |  | Ref |  | 0.486 |
| Q2 | 1.37 (1.12, 1.68) | 0.002 | 1.04 (0.75, 1.44) | 0.830 |  |
| Q3 | 1.84 (1.52, 2.25) | <0.001 | 1.55 (1.15, 2.10) | 0.004 |  |
| Q4 | 2.01 (1.65, 2.46) | <0.001 | 1.61 (1.20, 2.16) | 0.001 |  |
| CHG-BMI | | | | | |
| Q1 | Ref |  | Ref |  | 0.769 |
| Q2 | 1.33 (1.08, 1.63) | 0.007 | 1.35 (1.00, 1.82) | 0.049 |  |
| Q3 | 1.39 (1.13, 1.71) | 0.002 | 1.44 (1.07, 1.92) | 0.016 |  |
| Q4 | 1.85 (1.51, 2.26) | <0.001 | 1.73 (1.30, 2.31) | <0.001 |  |
| CHG-WC | | | | | |
| Q1 | Ref |  | Ref |  | 0.416 |
| Q2 | 1.37 (1.12, 1.68) | 0.002 | 1.30 (0.93, 1.80) | 0.120 |  |
| Q3 | 1.59 (1.30, 1.94) | <0.001 | 1.33 (0.97, 1.82) | 0.074 |  |
| Q4 | 1.96 (1.60, 2.39) | <0.001 | 1.53 (1.12, 2.08) | 0.007 |  |
| CHG-CVAI | | | | | |
| Q1 | Ref |  | Ref |  | 0.039 |
| Q2 | 1.68 (1.37, 2.06) | <0.001 | 1.61 (1.06, 2.43) | 0.025 |  |
| Q3 | 2.07 (1.69, 2.53) | <0.001 | 1.38 (0.92, 2.07) | 0.123 |  |
| Q4 | 2.55 (2.06, 3.14) | <0.001 | 1.90 (1.29, 2.80) | 0.001 |  |
| CHG-BRI | | | | | |
| Q1 | Ref |  | Ref |  | 0.253 |
| Q2 | 1.26 (1.03, 1.53) | 0.025 | 1.36 (0.97, 1.92) | 0.074 |  |
| Q3 | 1.59 (1.32, 1.93) | <0.001 | 1.47 (1.06, 2.04) | 0.023 |  |
| Q4 | 1.76 (1.44, 2.14) | <0.001 | 1.48 (1.07, 2.06) | 0.018 |  |
| CHG-WHtR | | | | | |
| Q1 | Ref |  | Ref |  | 0.236 |
| Q2 | 1.29 (1.06, 1.58) | 0.012 | 1.32 (0.93, 1.86) | 0.124 |  |
| Q3 | 1.63 (1.34, 1.98) | <0.001 | 1.51 (1.09, 2.11) | 0.015 |  |
| Q4 | 1.95 (1.59, 2.38) | <0.001 | 1.57 (1.13, 2.18) | 0.007 |  |
| CHG-WWI | | | | | |
| Q1 | Ref |  | Ref |  | 0.003 |
| Q2 | 1.30 (1.07, 1.58) | 0.008 | 1.11 (0.77, 1.59) | 0.577 |  |
| Q3 | 1.66 (1.37, 2.01) | <0.001 | 1.14 (0.81, 1.60) | 0.457 |  |
| Q4 | 2.07 (1.70, 2.53) | <0.001 | 1.18 (0.85, 1.65) | 0.328 |  |
| CHG-ABSI | | | | | |
| Q1 | Ref |  | Ref |  | 0.002 |
| Q2 | 1.21 (1.00, 1.47) | 0.055 | 1.09 (0.76, 1.54) | 0.646 |  |
| Q3 | 1.63 (1.35, 1.96) | <0.001 | 0.98 (0.70, 1.37) | 0.896 |  |
| Q4 | 1.84 (1.51, 2.25) | <0.001 | 1.09 (0.79, 1.51) | 0.604 |  |
| TyG | | | | | |
| Q1 | Ref |  | Ref |  | 0.161 |
| Q2 | 1.43 (1.17, 1.75) | <0.001 | 1.34 (0.99, 1.82) | 0.061 |  |
| Q3 | 1.75 (1.43, 2.13) | <0.001 | 1.48 (1.10, 1.98) | 0.009 |  |
| Q4 | 2.03 (1.66, 2.48) | <0.001 | 1.45 (1.08, 1.94) | 0.014 |  |
| CMI | | | | | |
| Q1 | Ref |  | Ref |  | 0.152 |
| Q2 | 1.41 (1.15, 1.73) | 0.001 | 1.29 (0.96, 1.74) | 0.097 |  |
| Q3 | 1.76 (1.44, 2.15) | <0.001 | 1.36 (1.02, 1.81) | 0.036 |  |
| Q4 | 1.95 (1.59, 2.38) | <0.001 | 1.41 (1.06, 1.89) | 0.020 |  |
| METS-IR | | | | | |
| Q1 | Ref |  | Ref |  | 0.211 |
| Q2 | 1.46 (1.18, 1.80) | <0.001 | 1.22 (0.90, 1.65) | 0.196 |  |
| Q3 | 1.82 (1.48, 2.24) | <0.001 | 1.38 (1.03, 1.85) | 0.029 |  |
| Q4 | 2.10 (1.71, 2.58) | <0.001 | 1.52 (1.14, 2.04) | 0.005 |  |
| AIP | | | | | |
| Q1 | Ref |  | Ref |  | 0.253 |
| Q2 | 1.45 (1.18, 1.78) | <0.001 | 1.34 (1.00, 1.80) | 0.047 |  |
| Q3 | 1.69 (1.38, 2.06) | <0.001 | 1.34 (1.01, 1.77) | 0.042 |  |
| Q4 | 1.99 (1.63, 2.43) | <0.001 | 1.51 (1.14, 2.01) | 0.004 |  |
| SHR, subdistribution hazard ratio; CI, confidence interval; Q1–Q4, quartiles.  Fine–Gray competing-risk regression with non-cardiovascular death as the competing event (n = 247).  Adjusted for sex, residence, smoking status, drinking status, marital status, education level, hypertension, hyperuricemia, and CKD (age excluded because it is the stratifying variable).  *P* for interaction was derived from a Wald test for the age × quartile interaction terms. | | | | | |
